# MicroRNA-9 promotes axon regeneration of mauthner-cell in zebrafish via *her6*/ calcium activity pathway

**DOI:** 10.1007/s00018-024-05117-2

**Published:** 2024-02-27

**Authors:** Yueru Shen, Xinghan Chen, Zheng Song, Huaitong Yao, Along Han, Yawen Zhang, Yuan Cai, Bing Hu

**Affiliations:** 1 Hefei National Research Center for Physical Sciences at the Microscale, University of Science and Technology of China, Hefei, 230026 China; 2 Center for Advanced Interdisciplinary Science and Biomedicine of IHM, Division of Life Sciences and Medicine, University of Science and Technology of China, Hefei, 230026 China; 3 First Affiliated Hospital of USTC, Division of Life Sciences and Medicine, University of Science and Technology of China, Hefei, 230026 China

**Keywords:** miRNA-9, Axon regeneration, *her6*, Calcium imaging, Mauthner

## Abstract

MicroRNA (miRNA), functioning as a post-transcriptional regulatory element, plays a significant role in numerous regulatory mechanisms and serves as a crucial intrinsic factor influencing axon regeneration. Prior investigations have elucidated the involvement of miRNA-9 in various processes, however, its specific contribution to axon regeneration in the central nervous system (CNS) remains uncertain. Hence, the zebrafish Mauthner axon regeneration model was employed to manipulate the expression of miRNA-9 in single cells, revealing that upregulation of miRNA-9 facilitated axon regeneration. Additionally, *her6*, a downstream target gene of miRNA-9, was identified as a novel gene associated with axon regeneration. Suppression of *her6* resulted in enhanced Mauthner axon regeneration, as evidenced by the significantly improved regenerative capacity observed in *her6* knockout zebrafish. In addition, modulation of *her6* expression affects intracellular calcium levels in neurons and promoting *her6* expression leads to a decrease in calcium levels in vivo using the new NEMOf calcium indicator. Moreover, the administration of the neural activity activator, pentylenetetrazol (PTZ) partially compensated for the inhibitory effect of *her6* overexpression on the calcium level and promoted axon regeneration. Taken together, our study revealed a role for miRNA-9 in the process of axon regeneration in the CNS, which improved intracellular calcium activity and promoted axon regeneration by inhibiting the expression of downstream target gene *her6*. In our study, miRNA-9 emerged as a novel and intriguing target in the intricate regulation of axon regeneration and offered compelling evidence for the intricate relationship between calcium activity and the facilitation of axon regeneration.

**Graphical Abstract:**

miRNA-9 can promote intracellular calcium activity in neurons by inhibiting the expression of its downstream target gene *her6*, which in turn promotes axonal regeneration. 
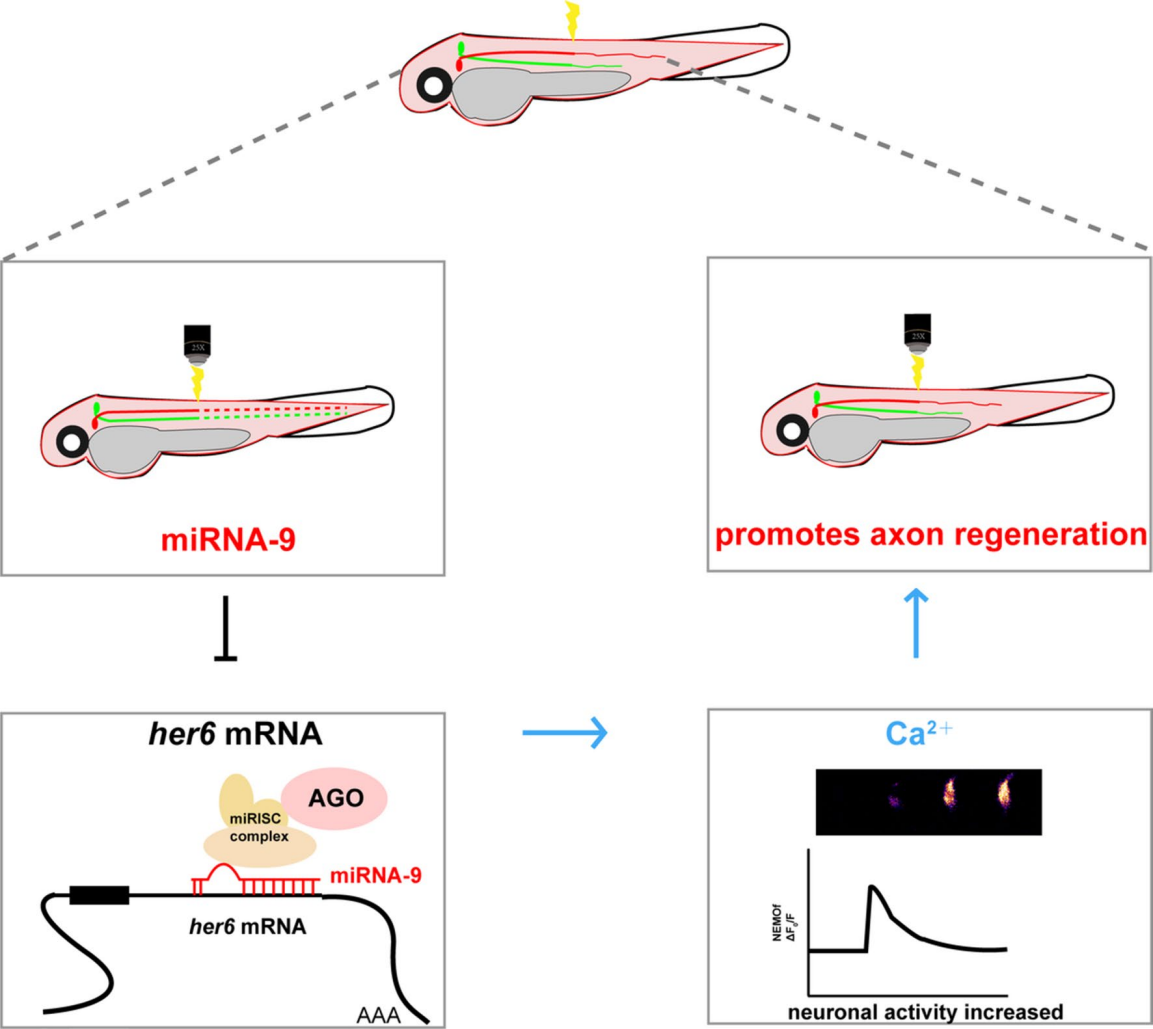

**Supplementary Information:**

The online version contains supplementary material available at 10.1007/s00018-024-05117-2.

## Introduction

Spinal cord injury (SCI) represents a highly disabling condition with a global prevalence, significantly affecting individuals at a young age, thereby exerting a profound impact on both the afflicted patients and their families [1]. Recent investigations have found that SCI are divided into primary spinal cord injury and secondary spinal cord injury [2]. This emerging insight forms a critical foundation for advancing our comprehension of this complex pathology and, ultimately, for devising innovative therapeutic strategies. The process of axon regeneration in the spinal cord is an important determinant in the functional recovery following SCI. Relative to the formidable challenges associated with axonal regeneration in the mammalian central nervous system (CNS), which often hinders recovery following injury, the peripheral nervous system (PNS) and the CNS axons of lower vertebrates, exemplified by the remarkable regenerative capabilities observed in species like the zebrafish, exhibit a remarkable ability to traverse the injury site and achieve regeneration, ultimately extending to reestablish nerve terminals. This difference in regeneration ability is usually divided into extrinsic factors and intrinsic factors [3]. Starting from the results that transplanting the central nervous into the environment of the peripheral axon can promote axon regeneration [4], some inhibitory factors for the axon regeneration process, such as scar glia and myelin inhibitory, have been found [2, 3, 5–7]. In recent years, researchers have focused more on the axon itself to explore the role of intrinsic factors in the process of axon regeneration. Accumulating evidence has substantiated the influence of various endogenous elements, including microtubule dynamics, microRNA (miRNA), and more, on the profound modulation of axon regeneration capabilities [8–11]. This burgeoning exploration furthered our understanding of the intrinsic mechanisms governing this intricate process.

MicroRNA (miRNA), as a pivotal epigenetic regulatory factor, primarily exerts its influence by inducing mRNA degradation or suppressing mRNA transcription through precise binding interactions with the 3' untranslated region (3'UTR) of target genes. It is estimated that the number of putative mRNA targets regulated by miRNAs at the post-transcriptional level is more than 60% of the total human genes [12]. It is thus evident that miRNAs assume an indispensable role in orchestrating post-transcriptional gene regulation. Increasingly, results show that miRNA emerges as a key orchestrator in a myriad of complex biological processes. Beyond its integral roles in axon guidance, glial cell proliferation, and differentiation, miRNA has influence extends to the intricate domains of tumorigenesis and axon regeneration [8, 13, 14]. The sequencing results of many miRNAs also indicate that abnormal expression of miRNA may be involved in the pathogenesis after SCI [8, 15]. Previous studies have shown that microRNA-9 (miRNA-9) regulates axon extension and branching by targeting Map1b in mice cortical neurons [16] and that miRNA-9 inhibits axon regeneration in peripheral sensory neurons [17]. Through the public data set of high-throughput sequencing data, we found that the expression of miRNA-9 and miRNA-135a were down-regulated after injury, and miRNA-135a was proved to promote axon regeneration and functional recovery after injury by targeting SP1 and ROCK [18]. Although these data suggest that miRNA-9 may be involved in many important events during axon development and regeneration, the role of miRNA-9 in CNS axon regeneration remains largely unknown. Particularly its regulatory role at the level of individual neurons is even less well understood.

The optical transparency of zebrafish makes it an important model animal for in vivo imaging research [19]. The Mauthner cells (M-cells) are a pair of motor neurons located in the hindbrain which belong to the CNS of the zebrafish. Compared with other single cells, the soma of the Mauthner cell is large, which facilitates observation and can be imaged in vivo at single-cell resolution. The axon extends from the hindbrain to the tail and is relatively independent, which confers distinct advantages to the observation of axon regeneration in vivo. It is worth mentioning that the Mauthner axon of zebrafish can basically regenerate completely at 96 h after injury [20]. Using the zebrafish Mauthner single-cell axon regeneration model, recent investigations have revealed that manipulating mitochondrial activity or neuronal activity in vivo can affect axon regeneration [21–24]. Characterization of changes in calcium levels in cells has been an important direction for detecting neuronal activity. GCaMP6f, GCaMP7f, and GCaMP8 have been commonly used as important probes for detecting calcium activity in neurons in recent years [25–27]. Combined with the advantages of zebrafish transparency, more and more studies have begun to use these probes for two-photon calcium imaging in zebrafish [21, 28–30]. NEMO is one of the newly developed genetically encoded calcium indicator (GECI) tools to monitor calcium signaling and cell activity in living cells in real-time. Studies have shown that NEMO has fast dynamics and a wide dynamic range, and the normalized basal brightness is much lower than that of GCaMP6f. Moreover, NEMO can be brighter without obvious photobleaching, which is more conducive to detecting weak calcium signals in the soma [31].

In the current study, we aimed to reveal the regulatory role of miRNA-9 in axon regeneration in the CNS and observe the direct binding of miRNA-9 to the downstream target gene *her6* by in vivo imaging. We then investigated the effect of modifying *her6* expression on the process of axonal regeneration and observed that such regulation results from changes in calcium dynamics by means of in vivo imaging. Taken together, our results indicated that miRNA-9 increased intracellular calcium levels and promoted neuronal activity by inhibiting its downstream target *her6*. This regulatory mechanism fosters the process of axon regeneration.

## Materials and methods

### Zebrafish transgenic lines and maintenance

AB/Wild type (WT) and Tg (Tol 056) strains with green fluorescent labeled M-cells were used in this study. All zebrafish lines were carefully maintained and raised at 28.5 ℃ under a 14/10 h light–dark cycle in the controlled aquatic habitat system. Embryos were transferred to a constant temperature incubator with 14 light and 10 dark after fertilization. After 24 hpf (hours post-fertilization), embryos were treated with 0.003% N-phenyl-2-thiourea (PTU, Sigma-Aldrich, USA) to prevent pigmentation.

All the animal manipulations were conducted in strict accordance with the guidelines and regulations set forth by the University of Science and Technology of China’s Animal Resources Center and University Animal Care and Use Committee. The protocol was approved by the Committee on the Ethics of Animal Experiments of the University of Science and Technology of China (permit USTCACUC1103013).

### Construction of plasmid

As a plasmid for overexpression effect, we amplified the genomic region of pri-miRNA-9 or the CDS sequence of *her6* and inserted it into the 3′ UTR of the UAS-mCherry plasmid using homologous recombination.

Based on the structure of miRNA, we constructed a plasmid named miRNA9-sponge. The six duplicates miRNA-9 reverse complementary sequences were synthesized by Songon (shanghai, China) and inserted into UAS-mCherry plasmid.

To knock down the expression of *her6*, we designed shRNA targeting *her6* sequence using the website: https://www.thermofisher.cn/. shRNA was amplified and cloned to the plasmid of UAS-mCherry-mir30e [22, 32, 33]. All primers used are listed in Supplementary Table S1.

### Microinjection and quantitative real-time polymerase chain reaction (qPCR)

On the night before spawning, the paired adult zebrafish are placed separately on both sides of the breeding tank, and the divider is opened at 8:30 a.m. on the spawning day to start mating. One-cell stage embryos were placed on a fluted agarose plate containing a small amount of EM solution. Approximately 1 nL of the mix solution or mRNA was injected per embryo.

Total miRNA was isolated from the 4 dpf (day post fertilization) larvae which expressed red fluorescence using the miRNA Isolation Kit (Tiangen, DP501, China), according to the manufacturer’s protocols. RNA concentration was measured by Nanodrop (Thermo Scientific, Waltham, USA). 1 μg miRNA was reversely transcribed into cDNA with miRNA First-Strand cDNA Kit (Tiangen, KR211, China) and cDNA was quantified by qPCR system (Light-Cycler 96, Roche) with miRNA qPCR Kit (Tiangen, FP411, China).To detect RNA levels, 4 dpf larvae with red fluorescence were selected to isolate total RNA using TRIzol (TARAKA, China), and the RNA was reverse-transcribed into cDNA with HiScript II qRT SuperMix II (Vazyme, China). The mRNA expression levels were quantified by the qPCR system using AceQ qPCR SYBR Master Mix (Vazyme, China). Twenty-five larvae were collected for each experimental condition. Each experiment was repeated three times in biology and technology. The qPCR primers used are listed in the Supplementary Table S1.

### Generation of transgenic zebrafish lines

Generation of miRNA-9 and *her6* knockout zebrafish lines by CRISPR/Cas9 gene editing. Single guide RNAs (sgRNAs) targeting miRNA-9 or *her6* for CRISPR/Cas9-mediated knock-out, were designed by the website: https://www.zlab.bio/ and synthesized with the Megashortscript T7 kit (Thermo Fisher). The Cas9 protein and sgRNA were mixed and injected into zebrafish embryos, and the final concentration of 300–500 ng/µL.

### Single-cell electroporation

The single-cell electroporation experiment of M-cells was conducted at 4 dpf of zebrafish larvae [34]. The larvae were anesthetized with ethyl 3-aminobenzoic methanesulfonate (MS222, Sigma-Aldrich) before the experiment, then fixed on a self-made agarose plate with 1% low-melting point agarose, and the plasmid in the glass electrode was electroporated into zebrafish unilateral M-cell with a 15V pulse lasting 500 ms.

### Two-photon axotomy

We performed the two-photon laser axotomy experiment on 6 dpf larvae. After fixation of MS222 anesthetized zebrafish larvae in 1% low-melting agarose, LSCM980 microscope (Carl Zeiss, Oberkochen, Germany) with a two-photon system was used to accurately damage the unilateral axon of the zebrafish Mauthner located above the cloacal pore [20, 35]. The two-photon laser used in this study was 800 nm and the power was 20–30% [34].

### In vivo imaging axon regeneration and body length

The in vivo imaging of Mauthner’s axon regeneration involved in this experiment was performed using a confocal microscope FV1000 (Olympus, Japan), using a 40 × hydroscope to measure the length of Mauthner’s axon from the cloaca pore to the terminal at 8 dpf [2 days post axotomy (dpa)]. The pictures of each region were obtained by Z-axis overlay and then the pictures were spliced by Adobe Photoshop CS4 (Adobe, San Jose, CA, USA).

Images of zebrafish body length were taken using a SZX16 (Olympus, Japan) microscope with 2 × magnification, which defined the total length of the zebrafish from head to tail as body length. Measurements were made using the ImageJ (National Institutes of Health, Bethesda, MD, USA). The zebrafish were anesthetized throughout the experiment.

### EGFP sensor assay

The EGFP-*her6* 3′-UTR, EGFP-*her6* mut-3′-UTR, and mCherry mRNA used in this experiment were transcribed in vitro using Thermo Fisher's kit, and then the mixtures of EGFP-*her6* 3′-UTR or EGFP-*her6* mut-3′-UTR and mCherry mRNA were injected into single cell of embryos according to the microinjection operation described above, respectively, to which equal amounts of 10 µM of miRNA-9 duplex as the experimental group and negative control (NC) as the control group. The larvae with red fluorescence were screened out after 24 h for imaging using a BX16 microscope (Olympus, Japan), and the fluorescence intensity of the larvae was measured by ImageJ.

### In vivo imaging of evoked calcium responses

Calcium response imaging of M-cells was performed using a confocal microscope FV1000, with a 40 × hydroscope lens to record the calcium signal intensity of M-cells treated with no anesthetic at 8 dpf (2 dpa). Scans were started at 330 ms intervals at the maximum plane of the cell body, a total of 100 images were scanned. A combined pulse (a series of 10 V, 5 ms wide bipolar pulses followed by a series of 10 V, 5 ms wide negative pulses) was applied to the area near the M-cell body halfway through the scan. The calcium signal intensity was measured using ImageJ (NIH, USA).

### Drug treatment

Pentylenetetrazol (PTZ) is a CNS stimulant that acts as a non-competitive antagonist of the GABA (A) receptor. Axon axotomy was performed in two groups of zebrafish receiving either EM water (as control group) or 2 mM PTZ (Selleck, China) (as experimental group) at 6 dpf. It was added in the fish water tank every 24 h at a final concentration of 0.1 μM from the day post-injury until 2 days post-injury according to the protocol in Fig. S6a. Imaging the regeneration length after 2 dpa.

### Free-swimming behavioral test

Behavioral tests of free-swimming in zebrafish were performed to test whether zebrafish motor function was affected by gene knockout. The viewpoint (Viewpoint, Lyon, France) instrument was used to record the free-swimming trajectory of 6 dpf and 8 dpf larvae for one hour, and the moving distance was calculated.

### Escape behavior assay

The functional recovery of zebrafish Mauthner neurons was examined by escape response test. The equipment used in this experiment included a high-speed photographic camera (1000 fps, Revealer, China), a sound source (produce 500 Hz, 20 ms sound stimulation), a lighting platform and a computer. Escape responses were tested on zebrafish larvae at 8 dpf which were placed in 60 mm diameter petri dishes containing EM solution. High-speed photographic cameras, adjusted to the suitable focal length, were employed to capture the responses. A computer-controlled the acoustic stimulus and recorded the process of the escape response. The videos were analyzed with professional software (Revealer, China) to record the maximum turn of larvae and the time required to reach the maximum turn when the escape response occurred.

### Statistical analysis

Graphs were proceeded with Adobe Illustrator CS6 (Adobe, San Jose, CA, USA). All data were presented in means ± standard error of the mean (SEM), and all analyses were performed by t-tests, one-ANOVAS tests, and non-parametric tests, where each experiment was repeated at least 3 times. All experimental data were presented in Excel (Microsoft, USA) and analyzed for significance using Prism 8 (San Diego, USA). *p ≤ 0.05, **p ≤ 0.01, ***p ≤ 0.001, ****p ≤ 0.0001.

## Results

### Conservation of miRNA-9 among species and developmental expression and characteristics in zebrafish

MiRNA-9 is an earlier type of miRNA studied, and previous studies mainly focused on its role in tumors [14]. With the development of sequencing technologies, miRNA-9 was found to be one of the most abundant miRNAs in the developing and adult brain [36, 37]. It has been found that miRNA-9 can regulate the proliferation of neural progenitor cells (NPCs) and participate in neurodegenerative diseases such as Huntington’s disease and Alzheimer’s disease [14].

**Fig. 1:**
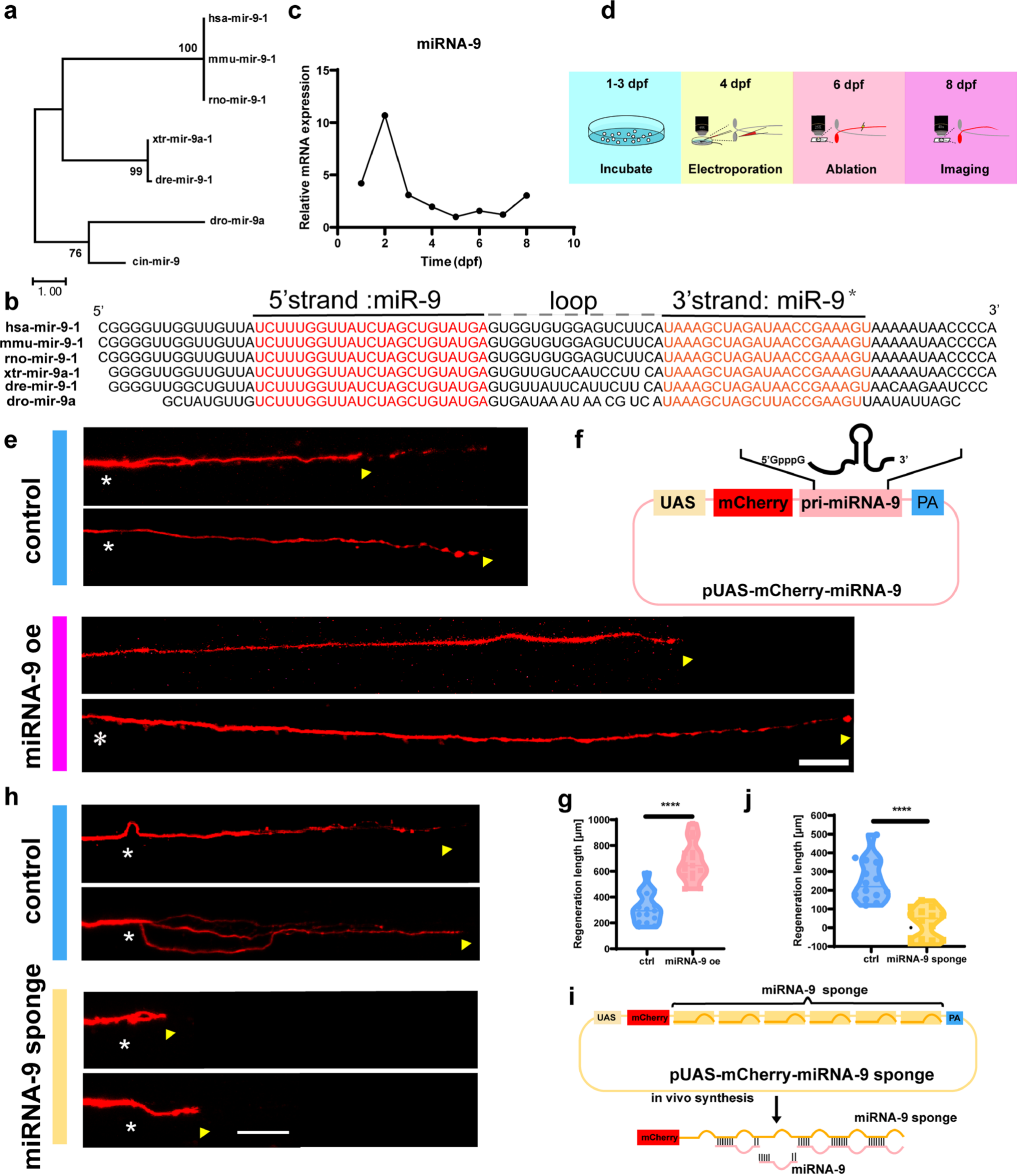
miRNA-9 regulates Mauthner-cell axon regeneration in vivo. **a** The phylogenetic tree with the maximum-likelihood algorithm of pri-miRNA-9-1 in humans, mice, rats, Xenopus, zebrafish, and Desmodus rotundus. The number represents the degree of homology, and miRNA-9-1 in zebrafish is highly homologous to miRNA-9-1 in humans. **b** Sequence alignment of pri-miRNA-9-1 in humans, mice, rats, Xenopus, zebrafish and Desmodus rotundus. Sequences and names were obtained from miRbase. The nucleotide sequences marked in red and orange are the mature miRNA-9, where red is the preferred strand and orange is the non-preferred strand. **c** Expression of miRNA-9-1 during 0-10 dpf development in Zebrafish. **d** Timeline of time points of electroporation, axotomy, and regeneration imaging. **e** Representative regeneration images of the M-cell axon with miRNA-9 overexpression. White asterisk, injury site; arrowhead, axon regeneration terminal; scale bar, 50 μm. **f** Construction of the miRNA-9 expression system. Plasmids express only mCherry served as the control vector. **g** miRNA-9 overexpression promotes M-cell axon regeneration (control: 313.3 ± 31.04 μm, n = 15 fish; miRNA-9 oe: 672.1 ± 38.87 μm, n = 15 fish). *p* < 0.0001. **h** Representative regeneration images of the M-cell axon with miRNA-9 sponge. Asterisk, injury site; arrowhead, axon regeneration terminal; scale bar, 50 μm. **i** Design of miRNA sponges. The construction of miRNA sponges was manipulated by inserting multiple miRNA binding sites in the 3′UTR of the mCherry. Plasmids express only mCherry served as the control vector. **j** miRNA-9 knockdown inhibits M-cell axon regeneration (control: 250.8 ± 29.49 μm, n = 14 fish; miRNA-9 sponge: 25.17 ± 21.51 μm, n = 14 fish). White asterisk: ablation point; arrowhead, axon regeneration terminal. scale bar, 50 μm. *p* < 0.0001. Assessed by unpaired t-test

Therefore, the alignment of miRNA-9 sequences resulted in the creation of a phylogenetic tree with neighbor-joining and maximum-likelihood algorithms through MEGA-11 software. Sequence analysis revealed remarkable nucleotide-level conservation of miRNA-9 across Xenopus and Homo sapiens (Fig. 1a, b). Such a high degree of evolutionary conservation would imply that the physiological functions of miRNA-9 are similar across species. The results of in situ hybridization (ISH) also found that miRNA-9 was expressed in the CNS at 48, 72, and 96 h in the zebrafish [38]. In addition, we also detected the expression of miRNA-9 during the developmental stage and found that its expression was the highest at 48 hpf, and then gradually decreased (Fig. 1c).

### Overexpression of miRNA-9 can promote axonal regeneration

Previous experimental results have shown that zebrafish M-cells can partially regenerate within 48 h after injury, providing an important experimental model for studying factors that affect axon regeneration in the CNS [20]. To investigate the role of miRNA-9 in the axon regeneration process of M-cell in zebrafish, we constructed a plasmid containing pri-miRNA-9 sequence with red fluorescent protein (Fig. 1f). Following the experimental procedure (Fig. S1a), CMV-Gal4-VP16, UAS-mCherry or UAS-mCherry-pri-miRNA-9 plasmids were simultaneously injected into zebrafish embryo at the single-cell stage. Subsequently, zebrafish larvae displaying red fluorescent patterns in the CNS were screened at 3 dpf (Fig. S1b). The expression of miRNA-9 was detected at 4 dpf. Our qPCR data revealed a remarkable 29-fold increase in the expression levels in the miRNA-9 overexpression (oe) group in comparison to the control group (Fig. S1c).

After verification that the constructed plasmid significantly increased miRNA-9 expression, we used this vector system at the single-cell level. Plasmids of CMV-Gal4-VP16/ UAS-mCherry-pri-miRNA-9 were injected into zebrafish unilateral M-cell using single-cell electroporation at 4 dpf, and the plasmids of CMV-Gal4-VP16/ UAS-mCherry served as the control (Fig. 1d). Subsequently, the zebrafish larvae were screened out with unilateral M-cell expressing red fluorescent protein at 5 dpf (Fig. 1d and Fig. S1e, S1f), and the axon was ablated with a two-photon laser at 6 dpf (Fig. 1d and Fig. S1g). The axon regeneration was imaging in vivo at 8 dpf (2 dpa). According to the imaging results, we found that the axon regeneration length of the experimental group overexpressing miRNA-9 was double longer than that of the control group (control: 313.3 ± 31.04 μm, n = 15 fish; miRNA-9 oe: 672.1 ± 38.87 μm, n = 15 fish; Fig. 1e, g). Such results reveal the great potential of miRNA-9 in the process of axon regeneration. In conclusion, this result proved that overexpression of miRNA-9 can promote axon regeneration in zebrafish M-cells at the single-cell level.

### Impaired function of miRNA-9 inhibited axonal regeneration

After overexpression of miRNA-9 at the single-cell level, our subsequent objective was to investigate the consequences of inhibiting miRNA-9 expression on axon regeneration at the single-cell level. Previous studies have shown that RNA transcripts such as long noncoding RNAs and circular RNAs can act as endogenous miRNA sponges to reduce their expression in vivo by binding to miRNAs [22, 39, 40]. According to the principle of action of sponge, we designed a reverse complementary sequence of six repeated miRNA-9 mature sequences, inserted behind the UAS-mCherry plasmid (Fig. 1i). And the effectiveness of this plasmid in reducing miRNA-9 expression was measured by qPCR (Fig. S1d).

Subsequently, we expressed miRNA-9 sponge plasmid in M-cells, ablated the red fluorescent axon with a two-photon laser, and then observed the regeneration of the axon. In vivo imaging showed that the length of axon regeneration was significantly reduced after the expression of the miRNA-9 sponge plasmid (control: 250.8 ± 29.49 μm, n = 14 fish; miRNA-9 sponge: 25.17 ± 21.51 μm, n = 14 fish; Fig. 1h, j), underscoring the regulatory influence of miRNA-9 on this regenerative process.

**Fig. 2:**
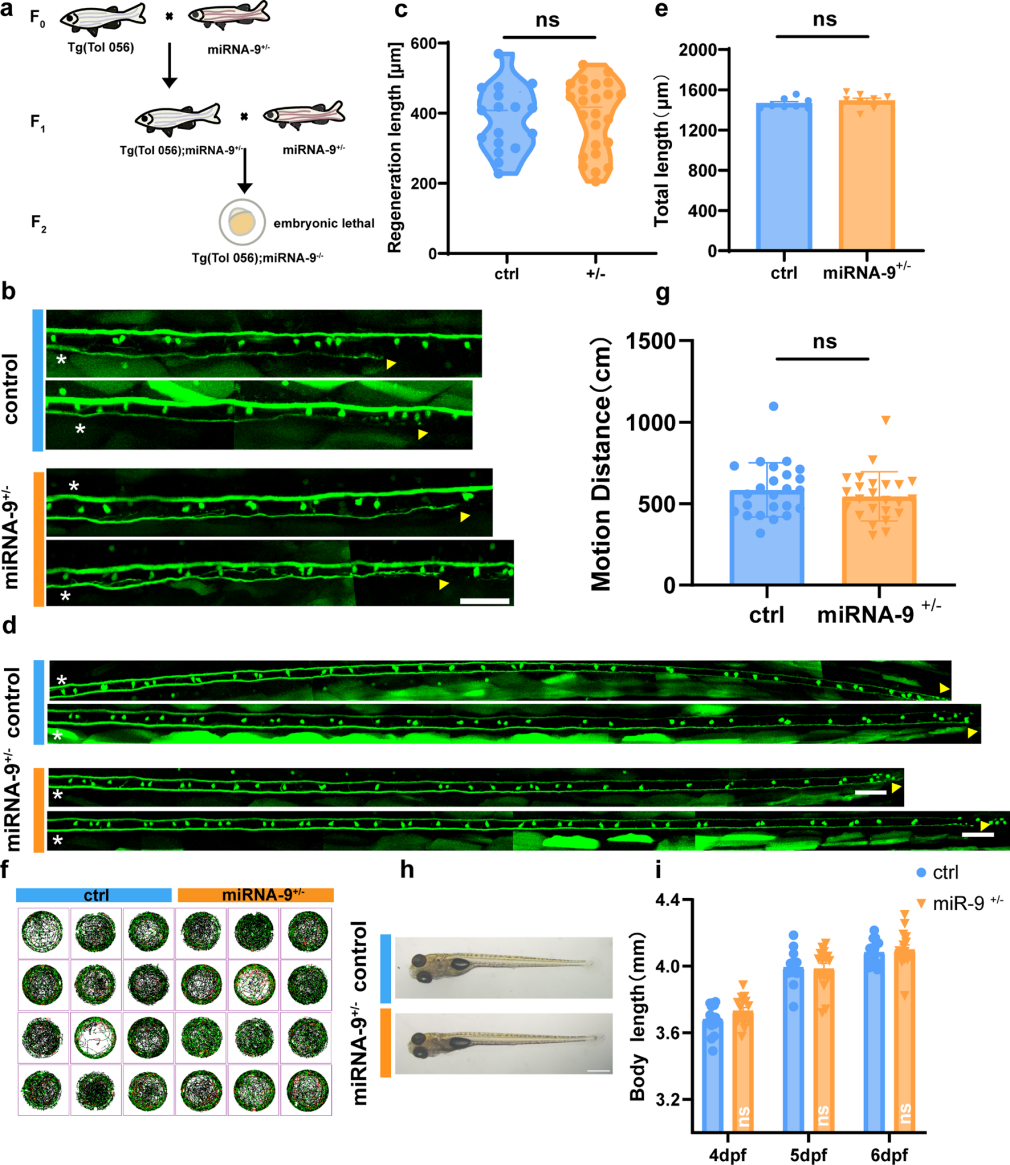
Heterozygote knockout of miRNA-9 did not affect M-cell axon regeneration and growth and development in zebrafish larvae. **a** Generation of miRNA-9^±^ and Tg (Tol-056); miRNA-9^+/−^ zebrafish. miRNA-9^−/−^ can cause embryonic lethality. **b**, **c** Heterozygote knockout of miRNA-9 did not affect M-cell axon regeneration (control: 383.4 ± 22.37 μm, n = 17 fish; miRNA-9^+/−^: 390.1 ± 20.69 μm, n = 24 fish). White asterisk: ablation point; arrowhead, axon regeneration terminal. scale bar, 50 μm. *p* = 0.8283, assessed by unpaired t-test, ns, not significant. **d**, **e** Total lengths of M-cell axons from the cloaca to the end were not notably different among WT and heterozygous knockout larvae (control: 1464 ± 17.8 μm, n = 8 fish; miRNA-9^+/−^: 1491 ± 26.07 μm, n = 8 fish). White asterisk: ablation point; arrowhead, axon regeneration terminal. scale bar, 50 μm. *p* = 0.4173, assessed by unpaired t-test, ns, not significant. **f**, **g** Trajectory diagrams and motion distance statistics of free swimming within 1 h of miRNA-9^+/−^ (control: 584.6 ± 34.03 cm, n = 24 fish; miRNA-9^+/−^: 545.3 ± 30.90 cm, n = 24 fish). *p* = 0.3973. Assessed by unpaired t-test. **h**, **i** Representative images of larvae from the wildtype and the miRNA-9^+/−^ at 6 dpf (scale bar, 500 μm), and measured total body length from 4 to 6 dpf (4 dpf: control: 3.68 ± 0.0226 mm; miRNA-9^+/−^: 3.730 ± 0.0217 mm, *p* = 0.1189, n = 15 fish; 5 dpf: control: 3.993 ± 0.0252 mm; miRNA-9^+/−^: 3.984 ± 0.0326 mm, *p* = 0.8324, n = 15 fish; 6 dpf: control: 4.082 ± 0.0192 mm; miRNA-9^+/−^: 4.098 ± 0.0293 mm, *p* = 0.6459, n = 15 fish). Assessed by two-way ANOVA. ns, not significant

In addition, we used CRISPR-Cas9 to generate a zebrafish strain with a targeted miRNA-9 knockout, aiming to observe the effect of miRNA-9 knockout on axon regeneration at the holistic level. Target design was performed on pri-miRNA-9 (Fig. S2a). In vivo 16 bp knockout zebrafish were obtained by microinjection of sgRNA and Cas9 protein (Fig. S2b-d), and it was found that the viable miRNA-9 homozygote knockout zebrafish could not be successfully obtained by hybridization (Fig. 2a). Therefore, we speculated that homozygote knockout of miRNA-9 may affect embryonic development in larvae. Subsequently, we observed axon regeneration on miRNA-9^+/−^ zebrafish. Interestingly, miRNA-9 heterozygote knockout did not affect axon regeneration (control: 383.4 ± 22.37 μm, n = 17 fish; miRNA-9^+/−^: 390.1 ± 20.69 μm, n = 24 fish; Fig. 2b, c). By observing the body length of miRNA-9^+/−^ individuals, it was found that there was no significant difference from the controls (4 dpf: control: 3.68 ± 0.0226 mm; miRNA-9^+/−^: 3.730 ± 0.0217 mm,* p* = 0.1189, n = 15 fish; 5 dpf: control: 3.993 ± 0.0252 mm; miRNA-9^+/−^: 3.984 ± 0.0326 mm, *p* = 0.8324, n = 15 fish; 6 dpf: control: 4.082 ± 0.0192 mm; miRNA-9^+/−^: 4.098 ± 0.0293 mm, *p* = 0.6459, n = 15 fish; Fig. 2h, i). And it has no significant effect on axon development (control: 1464 ± 17.8 μm, n = 8 fish; miRNA-9^+/−^: 1491 ± 26.07 μm, n = 8 fish; Fig. 2d, e). And it was also comparable in motor ability (control: 584.6 ± 34.03 cm, n = 24 fish; miRNA-9^+/−^: 545.3 ± 30.90 cm, n = 24 fish; Fig. 2f, g). Taken together, these findings implied that despite the miRNA-9 heterozygote knockout had not significant affect in the axon regeneration, the suppression of miRNA-9 expression in the single cell has the capacity to impede axon regeneration in zebrafish M-cells.

### *her6* was the in vivo target of miRNA-9

**Fig. 3:**
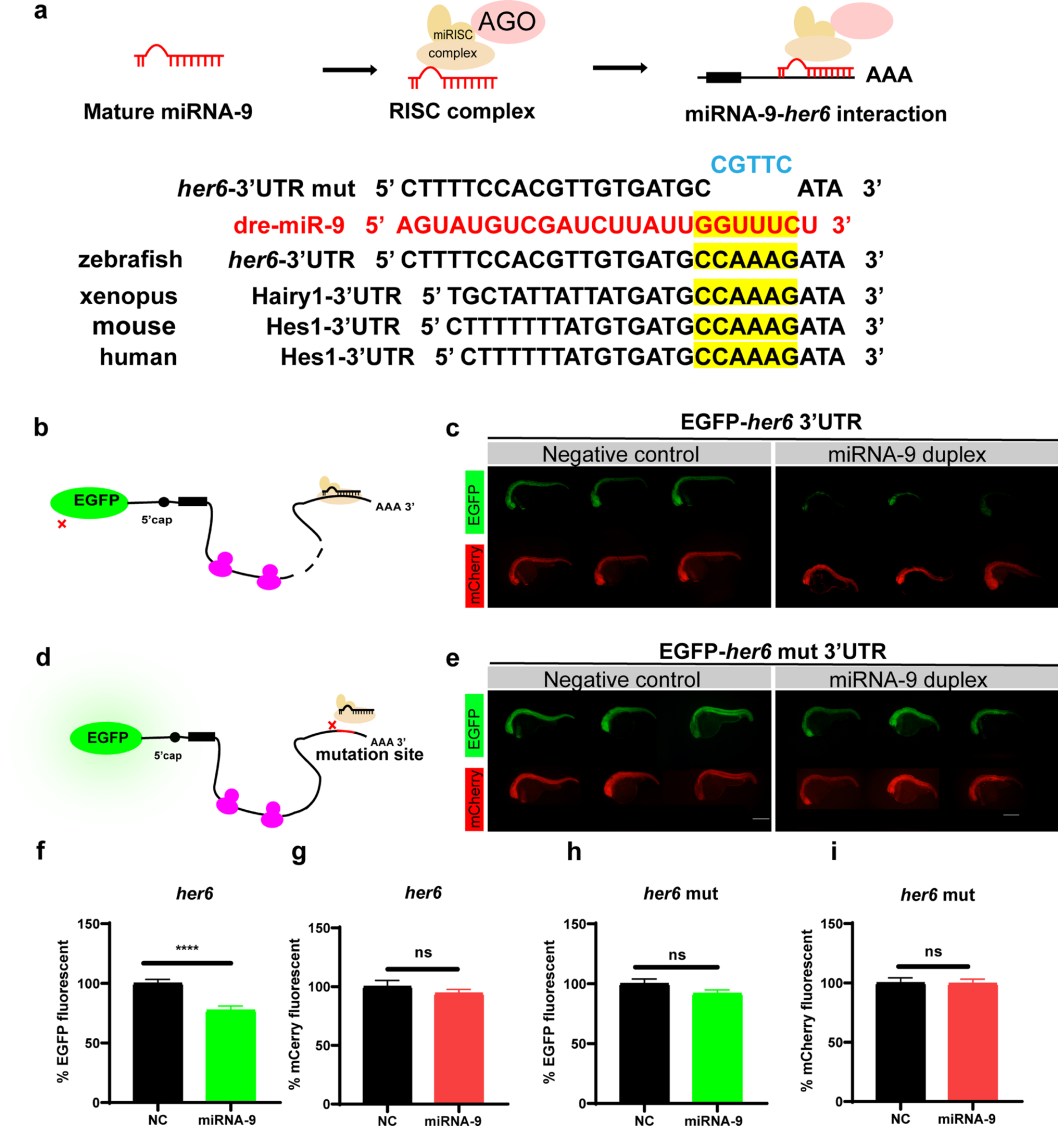
Sequence alignment and the EGFP sensor assay show that *her6* is the downstream target of miRNA-9. **a** Schematic representation of the interaction of miRNA-9 with *her6* and sequence comparison of miRNA-9, *her6* 3ʹUTR, and *her6* 3ʹUTR mutation (within the 2–7nt mutated). The zebrafish miRNA-9 mature sequence is shown in red, the seed sequence in yellow, and the mutant nucleotide sequence in blue. The homology of *her6* seed sequences in humans, mice, Xenopus, and zebrafish is also shown below. **b**, **c** EGFP-*her6* 3′UTR showed strong fluorescent signals when co-injected with non-sense duplex (as negative control), but failed to give fluorescent signals when co-injected with miRNA-9 duplex. mCherry mRNA was injected as a control. **d**, **e** EGFP-*her6* 3′UTR mut showed strong fluorescent signals both in the non-sense duplex and miRNA-9 duplex. **f**, **g** The EGFP (**f**) and mCherry (**g**) fluorescence was expressed as a percentage of the fluorescent signal observed from the negative control with the EGFP-*her6* 3′UTR. *p* < 0.0001 in EGFP fluorescence, *p* = 0.3404 in mCherry fluorescence, assessed by t-test. **h**, **i** The EGFP (**h**) and mCherry (**i**) fluorescence were expressed as a percentage of the fluorescent signal observed from the negative control with the EGFP-*her6* 3′UTR mut. *p* = 0.1070 in EGFP fluorescence, *p* = 0.9303 in mCherry fluorescence, assessed by t-test

Typically, the functionality of miRNA is manifest through its binding interactions with downstream targets, thereby instigating a complex cascade of molecular events. Through database analysis and previous research, we found that *her6* 3'UTR has the sequence complementary to the mature sequence of miRNA-9 and has 100% homology in vertebrates (Fig. 3a). Previous studies have shown that dynamic expression of miRNA-9 can influence *her* family expression [41, 42]. Her6 is a transcription repressor, belonging to the bHLH (basic helix-loop-helix)-orange family (Fig. S4a), which can bind to promoters and recruit corepressors to inhibit the effect of target genes. In most cases, *her6* assumes a pivotal role in the regulation of neurogenesis within the hindbrain region, concurrently engaging with the Notch signaling pathway, thereby orchestrating intricate processes critical for neural development. Therefore, we hypothesized that *her6* is the in vivo target of miRNA-9. To test this hypothesis, we first examined the expression level of *her6* after altering the expression of miRNA-9. Through the results of qPCR, it was proved that the expression level of *her6* mRNA was significantly downregulated after miRNA-9 overexpression, and upregulated after miRNA-9 sponge expressed (Fig. S3a, b).

Then we used EGFP-sensor to conduct in vivo imaging in zebrafish larvae. EGFP-sensor can observe the binding of miRNA to target in zebrafish in vivo, which is examined more visually by changes in fluorescent protein brightness [22, 43, 44]. We linked the *her6* 3ʹUTR sequence to the back of EGFP mRNA, with mCherry mRNA serving as an internal control in our experimental design. Fluorescence mRNA was injected with miRNA-9 duplex or negative control (NC) into the single cell stage of zebrafish embryo, and fluorescence intensity was observed 36 h later (Fig. 3b). Remarkably, the intensity of green fluorescent protein in the miRNA-9 duplex group exhibited a pronounced decrease, significantly lower than that observed in the negative control group (negative control: 100.0 ± 3.327%, n = 37 fish; miRNA-9 duplex: 77.32 ± 3.585%, n = 40 fish; Fig. 3c, f and Fig. S3c), while there was no large difference in red fluorescence intensity as an internal control (negative control: 100.0 ± 5.241%, n = 37 fish; miRNA-9 duplex: 94.28 ± 3.244%, n = 40 fish; Fig. 3c, g and Fig. S3c). Upon the introduction of mutations to the binding site within the *her6* 3ʹ UTR that interacts with miRNA-9 (Fig. 3a, d), our observations indicated the absence of any discernible difference in green fluorescence intensity between the miRNA-9 duplex and negative control groups (negative control: 100.0 ± 3.955%, n = 9 fish; miRNA-9 duplex: 91.73 ± 3.023%, n = 12 fish; Fig. 3e, h and Fig. S3c). Similarly, there was no significant difference in red fluorescence intensity (negative control: 100.0 ± 4.334%, n = 9 fish; miRNA-9 duplex: 99.50 ± 3.667%, n = 12 fish; Fig. 3e, i and Fig. S3c). Thus, the experimental results indicated that *her6* is the in vivo target of miRNA-9.

### *her6* affected the regenerative capacity of axons

**Fig. 4:**
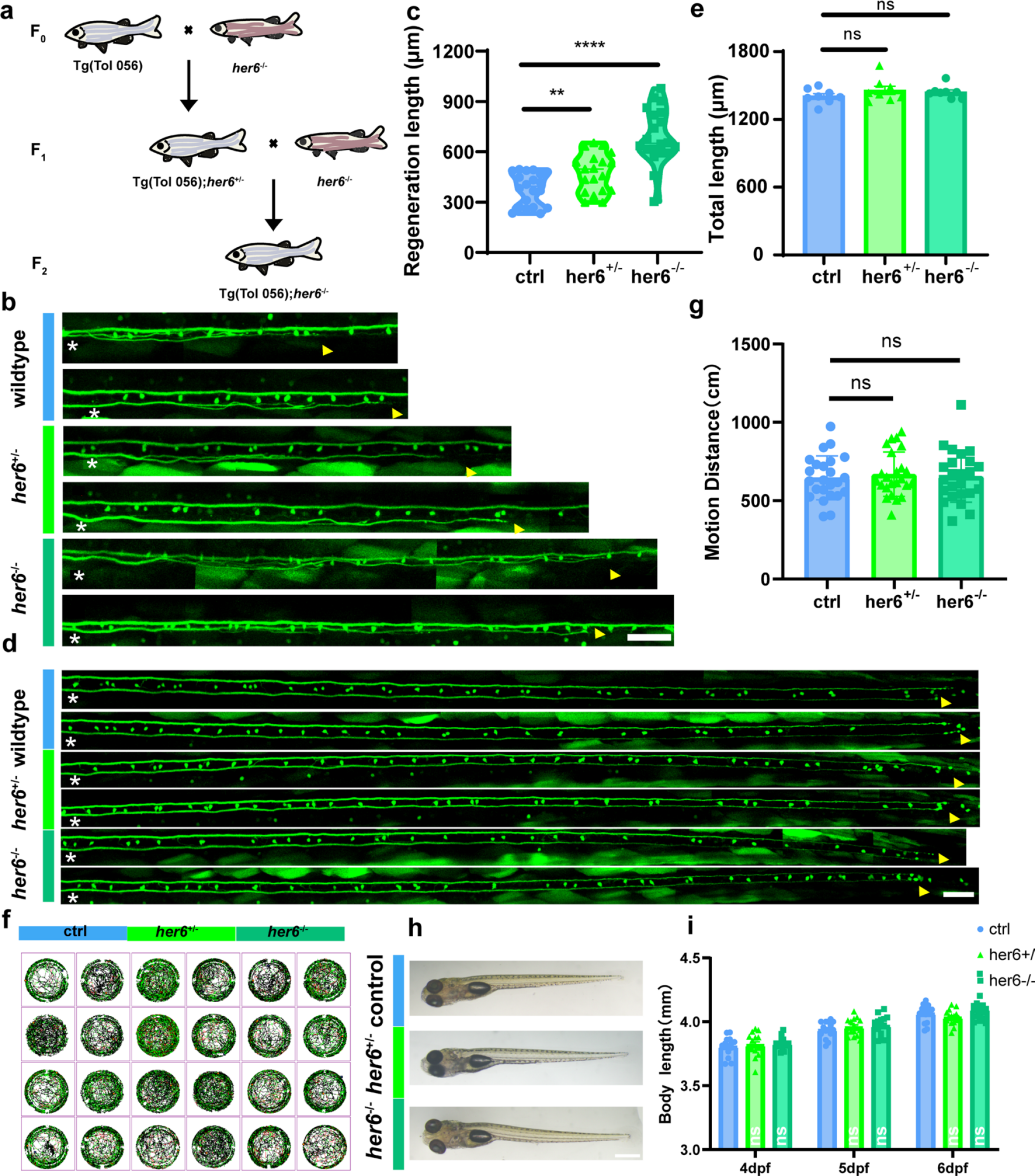
Deletion of *her6* facilitates M-cell axon regeneration and has no effect on growth and development in zebrafish larvae. **a** Generation of *her6*^−/−^ and Tg (Tol-056); *her6*^−/−^ zebrafish. **b**, **c** Homozygote knockout of *her6* promoted M-cell axon regeneration (control: 378.8 ± 21.57 μm, n = 20 fish; *her6*^+/−^: 476.6 ± 26.42 μm, n = 19 fish, *p* = 0.0066; *her6*^−/−^: 691.4 ± 36.86 μm, n = 20 fish; *p* < 0.0001). White asterisk: ablation point; arrowhead, axon regeneration terminal. scale bar, 50 μm. Assessed by unpaired t-test. **d**, **e** Total lengths of M-cell axons from the cloaca to the end were not notably different among WT, heterozygous and homozygote larvae (control:1406 ± 23.34 μm, n = 8 fish; *her6*^+/−^: 1458 ± 36.60 μm, n = 8 fish; *her6*^−/−^: 1441 ± 20.15 μm, n = 8 fish, *p* = 0.4140). White asterisk: ablation point; arrowhead, axon regeneration terminal. scale bar, 50 μm. *p* = 0.4140, assessed by one-way ANOVA, ns, not significant. **f**, **g** Trajectory diagrams and motion distance statistics of free swimming within 1 h of *her6*^−/−^(control: 645.8 ± 28.62 cm, n = 24 fish; *her6*^+/−^: 668.5 ± 28.90 cm, n = 24 fish; *her6*^−/−^: 654.2 ± 33.29 cm, n = 24 fish). *p* = 0.8674. Assessed by one-way ANOVA. ns, not significant. **h**, **i** Representative images of larvae from the wildtype, *her6*^+/−^ and *her6*^−/−^ at 6 dpf (scale bar, 500 μm), and measured total body length from 4 to 6 dpf (4 dpf: control: 3.807 ± 0.0193 mm, n = 15 fish; *her6*^+/−^: 3.820 ± 0.0238 mm, n = 15 fish; *her6*^−/−^: 3.831 ± 0.01201 mm, n = 15 fish, *p* = 0.6737; 5 dpf: control: 3.938 ± 0.0168 mm, n = 15 fish; *her6*^+/−^: 3.958 ± 0.0174 mm, n = 15 fish; *her6*^−/−^: 3.970 ± 0.0194 mm, n = 15 fish, *p* = 0.4588; 6 dpf: control: 4.066 ± 0.0185 mm, n = 15 fish; *her6*^+/−^: 4.034 ± 0.0153 mm, n = 15 fish; *her6*^−/−^: 4.098 ± 0.0251 mm, n = 15 fish, *p* = 0.0907). Assessed by two-way ANOVA, ns, not significant

After determining that miRNA-9 inhibits the expression of *her6*, we wanted to know whether *her6* would regulate axon regeneration. To explore the effect of *her6* on axon regeneration, we first designed a CRISPR-Cas9 target site at the fourth exon of *her6* (Fig. S4a, S4b), and synthesized the sgRNA by using the kit. After two generations of crosses (Fig. 4a), we obtained a *her6* systemic knockout zebrafish line with a 4 bp deletion that resulted in an early termination codon (Fig. S4c-S4f).

Next, we crossed the *her6*^−/−^ zebrafish with the transgenic strain Tg (Tol 056) to obtain Tg (Tol 056); *her6*^−/−^ zebrafish strain which was used to investigate the role of *her6* in zebrafish Mauthner axon regeneration. Axon axotomy was performed using a two-photon laser on zebrafish larvae at 6 dpf, followed by imaging of axon regeneration at 2 dpa. Based on the in vivo imaging results, we found that axon regeneration capacity was increased in the *her6*^+/−^ group, and *her6* complete knockout significantly increased axon regeneration length (control: 378.8 ± 21.57 μm, n = 20 fish; *her6*^+/−^: 476.6 ± 26.42 μm, n = 19 fish; *her6*^−/−^: 691.4 ± 36.86 μm, n = 20 fish; Fig. 4b, c).

To ascertain the effect of *her6* deletion on the overall growth and development of larvae, we calculated the body length after *her6* deletion. Notably, our observations indicated that *her6* deletion had no significant effect on the larvae in body growth and development (4 dpf: control: 3.807 ± 0.0193 mm, n = 15 fish; *her6*^+/−^: 3.820 ± 0.0238 mm, n = 15 fish; *her6*^−/−^: 3.831 ± 0.0121 mm, n = 15 fish; 5 dpf: control: 3.938 ± 0.0168 mm, n = 15 fish; *her6*^+/−^: 3.958 ± 0.0174 mm, n = 15 fish; *her6*^−/−^: 3.970 ± 0.0194 mm, n = 15 fish; 6 dpf: control: 4.066 ± 0.0185 mm, n = 15 fish; *her6*^+/−^: 4.034 ± 0.0153 mm, n = 15 fish; *her6*^−/−^: 4.098 ± 0.0251 mm, n = 15 fish; Fig. 4h, i). We also observed the free swimming of zebrafish, and noted that there was no notable disparity in the swimming distance of larvae within a one-hour interval between the *her6*-knockout group and the control group (control: 645.8 ± 28.62 cm, n = 24 fish; *her6*^+/−^: 668.5 ± 28.90 cm, n = 24 fish; *her6*^−/−^: 654.2 ± 33.29 cm, n = 24 fish; Fig. 4f, g), which indicated that *her6* deficiency did not affect the motor ability of zebrafish. In addition, we measured the total axon length of M-cells of larvae from the cloaca to the terminal, and found that there was no significant difference in the axonal development of M-cells at 6 dpf with *her6* deletion compared with the control group (control:1406 ± 23.34 μm, n = 8 fish; *her6*^+/−^: 1458 ± 36.60 μm, n = 8 fish; *her6*^−/−^: 1441 ± 20.15 μm, n = 8 fish; Fig. 4d, e). The above results proved that the systemic deletion of *her6* impairs the regeneration ability of axons, without affecting the growth and axonal development of larvae.

**Fig. 5:**
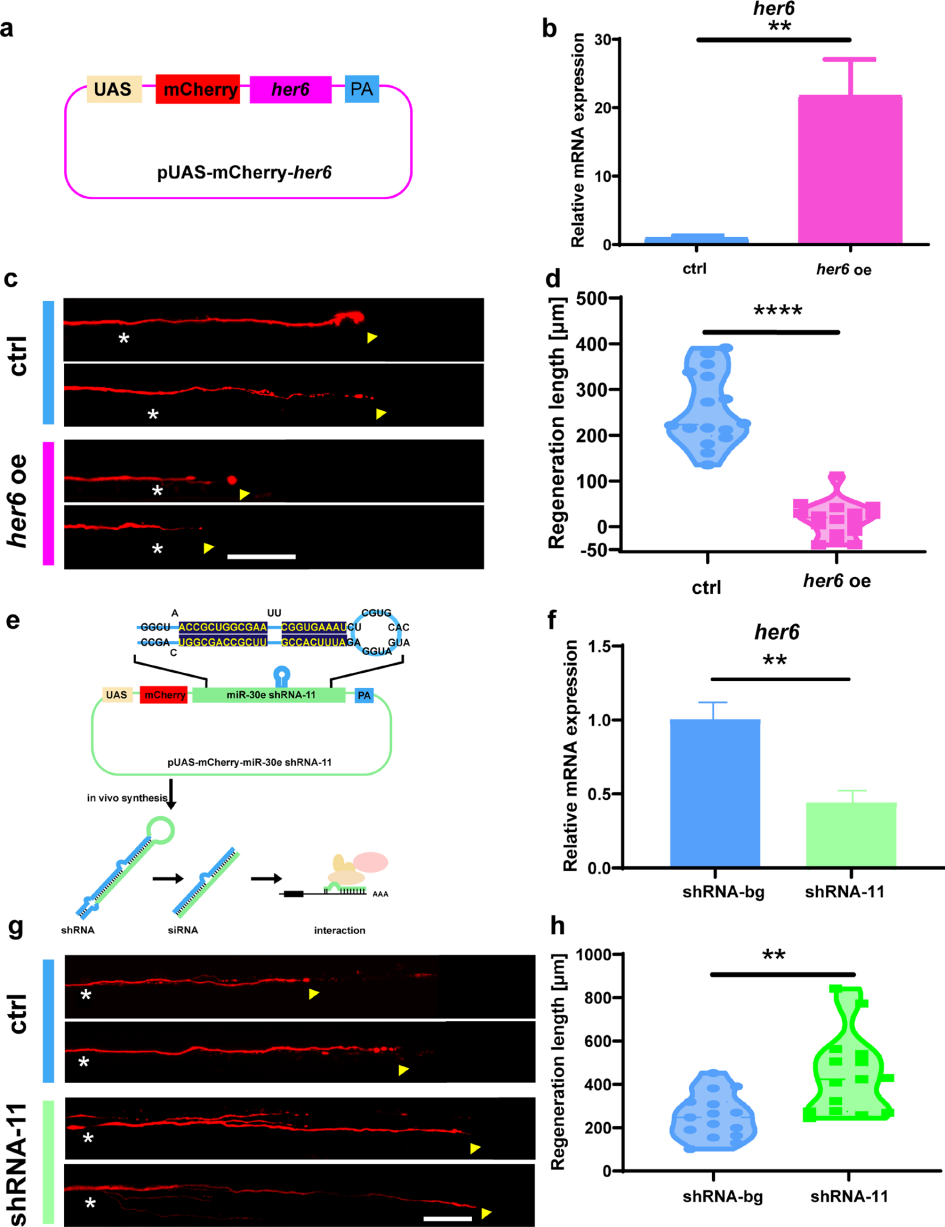
*her6* regulates Mauthner-cell axon regeneration in vivo. **a** Construction of the *her6* expression system. Plasmids express only mCherry served as the control vector. **b** Quantitative RT-PCR analysis exhibited overexpression of *her6* in 4 dpf zebrafish larvae by the vector-based *her6* oe in vivo. *p* = 0.0012. Assessed by unpaired t-test. **c**, **d** *her6* overexpression inhibits M-cell axon regeneration (control: 255.8 ± 19.62 μm, n = 16 fish; *her6* oe: 19.18 ± 9.603 μm, n = 16 fish). White asterisk: ablation point; arrowhead, axon regeneration terminal. scale bar, 50 μm. *p* < 0.0001. Assessed by unpaired t-test. **e** Design of the *her6* shRNA expression system based on the miR-30e backbone. Plasmids express only mCherry served as the control vector. **f** Quantitative RT-PCR analysis exhibited a reduction of *her6* in 4 dpf zebrafish larvae by the vector-based *her6* shRNA-11 in vivo. *p* = 0.0019. Assessed by unpaired t-test. **g**, **h** Decreased expression of *her6* inhibits M-cell axon regeneration (control: 253 ± 26.51 μm n = 15 fish; *her6* shRNA-11: 442.7 ± 47.6 μm, n = 15 fish). White asterisk: ablation point; arrowhead, axon regeneration terminal. scale bar, 50 μm. *p* = 0.0017. Assessed by unpaired t-test

Nonetheless, the above *her6* deletion represents a systemic effect, and the regulation effect of *her6* on axons in a single cell remains obscure, so we next regulated *her6* expression in single Mauthner cells. We up-regulated *her6* expression in Mauthner single cells with the overexpression plasmid (Fig. 5a, b), ablated axons expressing red fluorescence at 6 dpf, and performed imaging of axon regeneration at 2 dpa. Like the results of miRNA-9 sponge, overexpression of *her6* significantly inhibited axon regeneration (control: 255.8 ± 19.62 μm, n = 16 fish; *her6* oe: 19.18 ± 9.603 μm, n = 16 fish; Fig. 5c, d).

To inhibit *her6* expression, we used shRNA with miRNA-30e as the backbone and designed shRNAs 1–5 and 11 using the shRNA design tool (https://rnaidesigner.thermofisher.com/) (Fig. 5e and Fig. S5a–c). Subsequently, we tested the effectiveness of the shRNAs in the larvae (Fig. S5a–c). Intriguingly, we found that shRNA-11 was able to effectively reduce *her6* expression level in vivo (Fig. 5f). We next inhibited *her6* expression in single cells and observed its effect on axon regeneration later. The results revealed that *her6* shRNA-11 could significantly improve the regeneration capacity of Mauthner axons (control: 253 ± 26.51 μm, n = 15 fish; *her6* shRNA-11: 442.7 ± 47.6 μm, n = 15 fish; Fig. 5g, h). Combining the above results, we proved that *her6* plays a facilitating role in the process of axon regeneration.

### *her6* affected calcium activity in neurons

Calcium plays a pivotal role in orchestrating a myriad of intricate processes within the organism. It actively contributes to the transmission of neurotransmitters and serves as a key initiator of axonal regeneration[25–27, 29, 30]. The neuronal activity driven by calcium dynamics exerts a regulatory influence on axon regeneration, underscoring the significance of alterations in calcium levels as pivotal indicators throughout the process of axon regeneration [25–27, 29, 30]. Capitalizing on the unique transparency of zebrafish and their inherent capability for in vivo imaging, we employed the novel calcium probe, NEMOf, to capture calcium level dynamics during axon regeneration. This allowed us to scrutinize fluctuations in calcium levels under various regenerative conditions. NEMOf is a new calcium indicator designed based on mNG (mNeonGreen), which can detect changes in calcium levels in vivo in trace amounts [31].

**Fig. 6:**
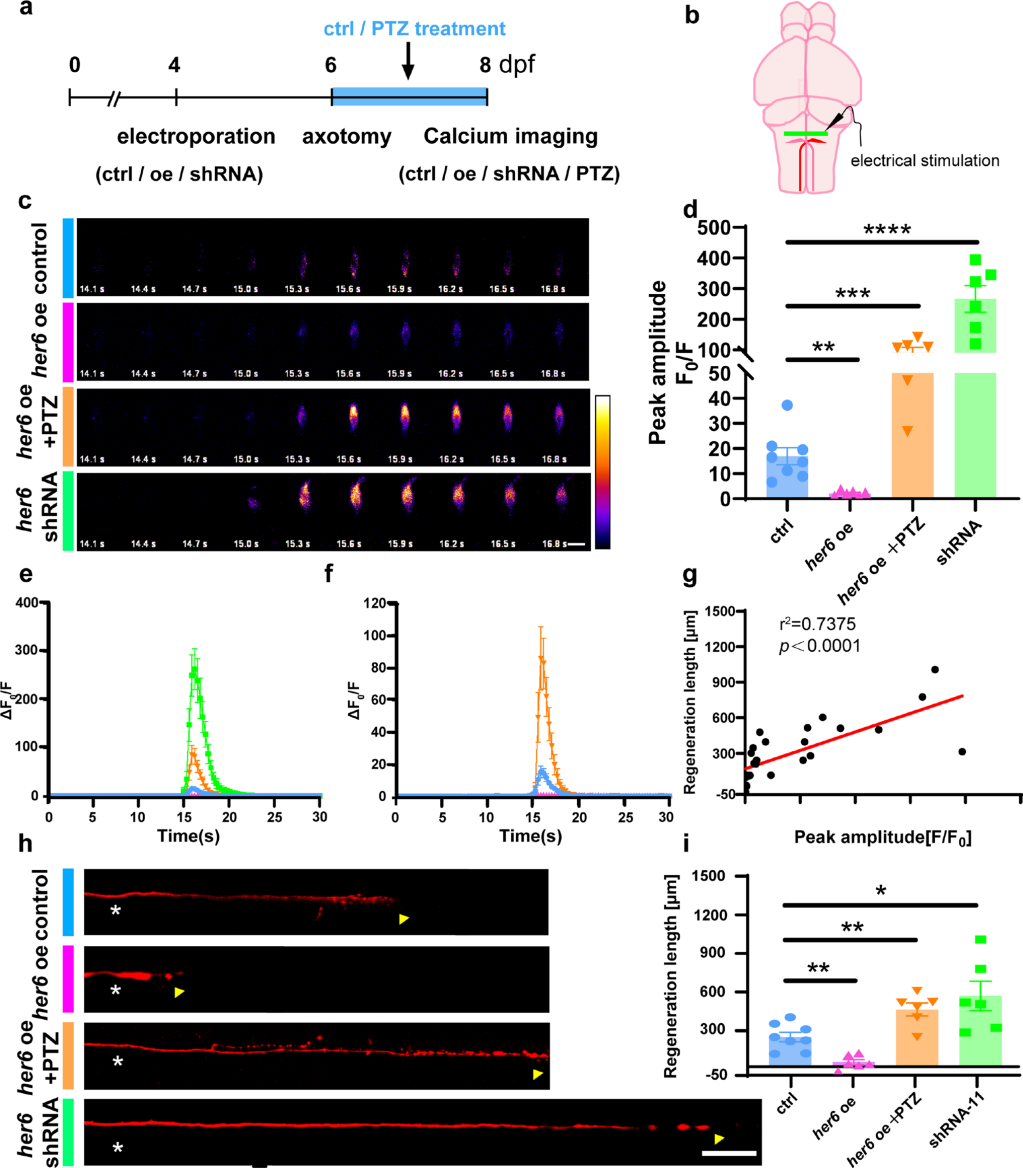
*Her6* regulates calcium activity in M-cells and affects axon regeneration in vivo*.* **a** The time axis showed the time-points of electroporation, axotomy, dynamic calcium imaging, and regeneration imaging. One of the pharmacological-treated groups was treated with PTZ for 48 h after injury and then imaging of calcium activity and regeneration was performed. **b** Diagram of the brain of zebrafish and the location of electrical stimulation in M-cells. **c** Representative examples of M-cell calcium activity after electrical stimulation in larvae labeled with NEMOf. The time after the stimulation (in seconds) is given in each frame. The color scale indicates fluorescence intensity (black: lowest; white: highest; Scale bar, 20 μm). **d** The peak amplitude of the calcium response traces in larvae that regulate *her6* expression by plasmid or pharmacologically (control: 16.96 ± 3.388, n = 8 fish; *her6* oe: 2.108 ± 0.4145, n = 6 fish; *her6* shRNA-11: 266 ± 43.45, n = 6 fish; *her6* oe + PTZ: 457.3 ± 51, n = 6 fish). Assessed by one-way ANOVA. **e**, **f** The calcium response elicited in larvae overexpressing *her6* was much weaker than that in control larvae and the response elicited in larvae *her6* shRNA was much stronger than that in control larvae. **g** The relationship between regeneration length and peak amplitude of calcium response has a significant positive correlation (r^2^ = 0.7375, n = 26). **h**, **i** Altered *her6* expression affects M-cells axon regeneration (control: 241.5 ± 36.38 μm, n = 8 fish; *her6* oe: 44.38 ± 21.65 μm, n = 6 fish; *her6* shRNA-11: 563.3 ± 114.2 μm, n = 6 fish; *her6* oe + PTZ: 457.3 ± 51 μm, n = 6 fish). White asterisk: ablation point; arrowhead, axon regeneration terminal. scale bar, 50 μm. Assessed by one-way ANOVA

We followed the protocol to electroporate the mixture of calcium probes and regulatory plasmids (UAS-mCherry; UAS-mCherry-*her6* oe; UAS-mCherry-*her6*-shRNA-11), laser ablation of the fluorescent protein-expressing Mauthner axons at 6 dpf, followed by detailed imaging to track and analyze changes in calcium levels in larvae of WT at 2 dpa (Fig. 6a). Significantly, for the detection of fluctuations in calcium levels, we introduced electrical stimulation proximity to the soma of Mauthner cells via glass electrodes (Fig. 6b). This stimulation effectively induced the expression of calcium probes, allowing us to monitor neuronal activity induced by calcium dynamics throughout 30 s. In terms of imaging results, we found that the change of calcium level in *her6* shRNA-11 was very dramatic, which was significantly stronger than that in the control group, while the change of calcium level in the experimental group overexpressing *her6* was weak, which was decreased significantly compared with the control group (Fig. 6c–f). In fact, the comparison of the peak amplitude of the calcium response also supports the above point (control:16.96 ± 3.38, n = 8 fish; *her6* oe: 2.11 ± 0.41, n = 6 fish; *her6* shRNA-11: 266.00 ± 43.45, n = 6 fish; Fig. 6d). Since the neural activity induced by calcium response has an impact on axon regeneration, we also measured the axon regeneration length in each group. As in the previous results, the length of regeneration was significantly shorter after overexpressing *her6* than in the control group, whereas the regeneration length in the experimental group in which *her6* expression was inhibited by shRNA-11 was significantly longer than that of the control group (control: 241.50 ± 36.38 μm, n = 8 fish; *her6* oe: 44.38 ± 21.65 μm, n = 6 fish; *her6* shRNA-11: 563.30 ± 114.20 μm, n = 6 fish; Fig. 6h, i). Meanwhile, through linear correlation analysis, we unveiled a statistically significant positive correlation between regeneration length and peak amplitude of calcium response (*r*^2^ = 0.7375, Fig. 6g). Altogether, *her6* affected neural calcium activity in M-cells.

### Altered *her6* expression induced changes in calcium levels affected Mauthner axon regeneration

Pharmacological interventions offer an additional avenue to modulate neuronal activity, complementing the regulatory potential of plasmid expression. Pentylenetetrazole (PTZ), a central nervous system stimulant, is commonly used to construct epilepsy models in mice and zebrafish, which affects neuronal activity and calcium signaling in vivo [21, 45–47]. Therefore, we first performed a pharmacological treatment of PTZ on zebrafish after 6 dpf injury to observe whether axonal regeneration would be affected by PTZ treatment (Fig. S6a). According to the statistics of axonal regeneration length, we found that the length of the drug treatment group was significantly longer than that of the control group (control: 325.50 ± 21.72 μm, n = 23 fish; PTZ: 505.00 ± 16.76 μm, n = 32 fish; Fig. S6b, S6c).

Then we wanted to observe whether PTZ treatment had the potential to alleviate the effects of *her6* overexpression on calcium levels and axon regeneration. We ablated the axons of zebrafish with *her6* overexpression in single cells at 6 dpf, then treated them in 2 mM PTZ, and observed the changes in the calcium level of M-cells at 2 dpa (Fig. 6a). The results showed that PTZ treatment has a significant improvement in the neuronal calcium response, and the peak amplitude had significantly increased (*her6* oe + PTZ:90.27 ± 17.86, n = 6 fish; Fig. 6c–f). As expected, the axonal regeneration ability was restored significantly in the *her6* overexpression group after PTZ treatment (*her6* oe + PTZ: 457.3 ± 51 μm, n = 6 fish; Fig. 6h, i). On this basis, we conclusively demonstrated that *her6* has the capacity to induce alterations in calcium levels, subsequently influencing the process of axon regeneration.

### Axon regeneration induced by *her6*^*−/−*^ promotes recovery of motor function in zebrafish

The escape response in zebrafish is a rapid evasion behavior typically triggered in response to external sound stimuli or dangerous objects such as natural enemies. And the key neurons that drive the escape response are Mauthner cells [48, 49]. Mauthner neuron cell bodies located in the brain can sense stimuli and transmit signals to the spinal cord, thereby driving muscles to move away from the stimulus [50, 51]. Certainly, the escape response in zebrafish encompasses two distinct behaviors: C-start, which is propelled by the Mauthner cells, and the escape swimming behavior. Previous studies have established a notable correlation between the recuperation of Mauthner cells and the C-start response [48–51]. Our above results revealed that *her6*^*−/−*^ zebrafish had a significant increase in axon regeneration (Fig. 4b, c), while discernible alterations were not evident in their free-swimming behavior at 8 dpf (2 dpa) (Fig. S7a, 7b). Consequently, we aimed to examine the functional recovery of *her6*^*−/−*^ larvae through escape response.

**Fig. 7:**
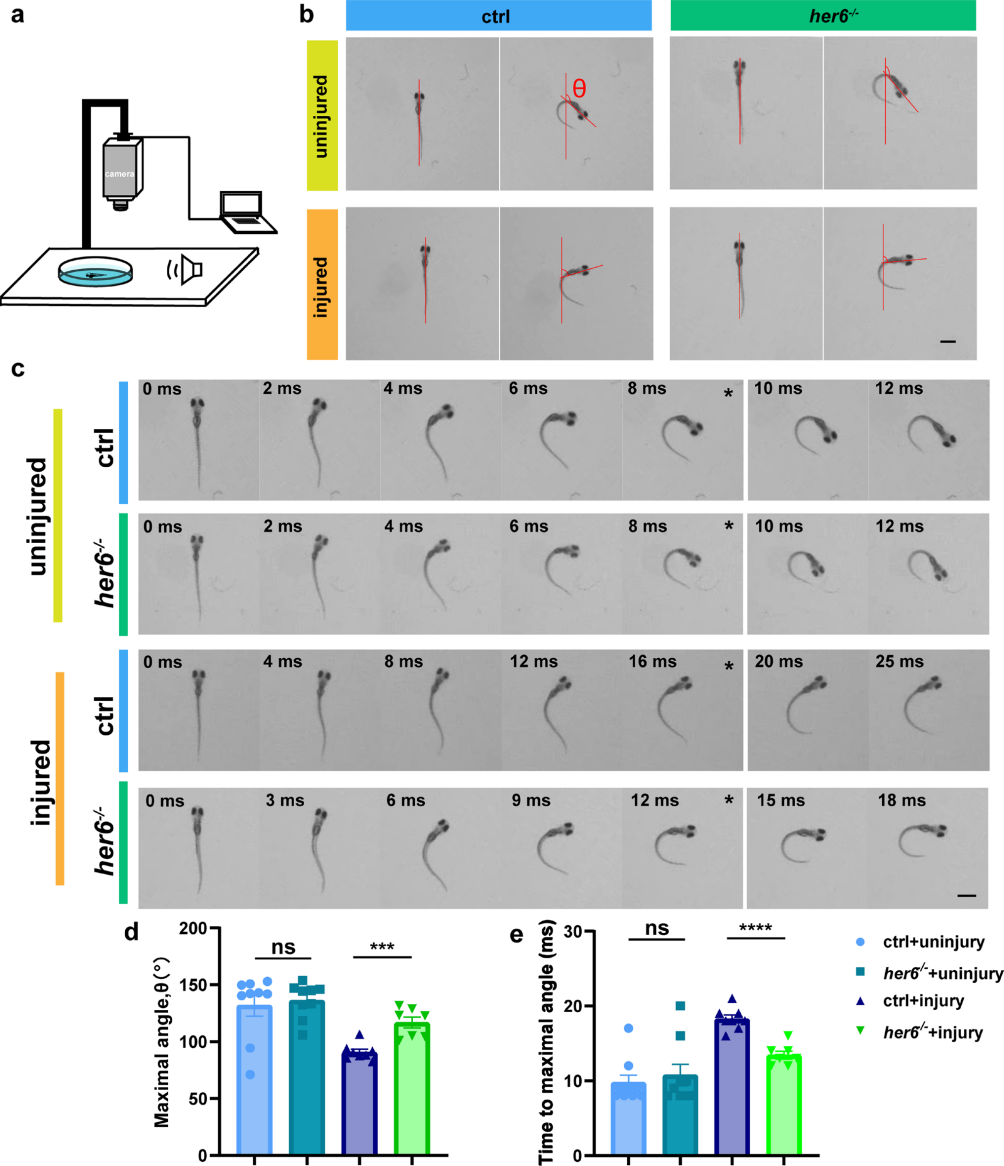
Axon regeneration induced by *her6*^*−/−*^ promotes recovery of motor function in zebrafish. **a** Diagram of the equipment to introduce and record the escape response. **b** Representative images of the initial position and maximal turn angle position from the control and *her6*^*−/−*^ zebrafish larvae in the uninjured and injured groups. The red line represents the direction of the current position. θ represents the maximal angle of rotation of the head. scale bar, 1 mm. **c** A series of images of escape response in the control and *her6*^*−/−*^ zebrafish larvae in the uninjured and injured groups. Time after the escape response (in milliseconds) is given in each frame. * represents the time for the maximum turning angle scale bar, 1 mm. **d** The maximum turn angle of escape in the *her6*^*−/−*^ larvae was significantly higher than that in the control group after injury. (control + uninjured: 131.8 ± 9.591°, n = 9 fish; *her6*^*−/−*^ + uninjured: 136.2 ± 5.193°, n = 9 fish; control + injured: 90.8 ± 2.569°, n = 8 fish; *her6*^*−/−*^ + injured: 116.7 ± 4.823°, n = 7 fish). Assessed by one-way ANOVA. **e** The time required to reach the maximum turning angle in the *her6*^*−/−*^ larvae was significantly shorter than that in the control group after injury. (control + uninjured: 9.778 ± 0.9969 ms, n = 9 fish; *her6*^*−/−*^ + uninjured: 10.78 ± 1.4320 ms, n = 9 fish; control + injured: 18.25 ± 0.5261 ms, n = 8 fish; *her6*^*−/−*^ + injured: 13.41 ± 0.5281 ms, n = 7 fish). Assessed by one-way ANOVA

We used a high-speed camera to record the escape response of zebrafish after being stimulated by sound (Fig. 7a). By analyzing the maximum turn angle and the time required to reach the maximum turning angle, we found that there was no significant difference in the maximum turning angle of escape and the time to reach the maximum turning angle in the uninjured control group and the *her6*^*−/−*^ group at 8 dpf. In contrast, the maximum turn angle of escape in the *her6*^*−/−*^ group was significantly higher than that in the control group after injury (control + uninjured: 131.8 ± 9.591°, n = 9 fish; *her6*^*−/−*^ + uninjured: 136.2 ± 5.193°, n = 9 fish; control + injured: 90.8 ± 2.569°, n = 8 fish; *her6*^*−/−*^ + injured: 116.7 ± 4.823°, n = 7 fish, Fig. 7b–e), and the time required to reach the maximum turning angle in the *her6*^*−/−*^ group was also significantly shorter (control + uninjured: 9.77 ± 0.9969 ms, n = 9 fish; *her6*^*−/−*^ + uninjured: 10.78 ± 1.4320 ms, n = 9 fish; control + injured: 18.25 ± 0.5261 ms, n = 8 fish; *her6*^*−/−*^ + injured: 13.41 ± 0.5281 ms, n = 7 fish, Fig. 7b–e). These results indicate a clear functional restoration of the escape response driven by Mauthner cells in the *her6*^*−/−*^ zebrafish.

## Discussion

Crucially, within both central and peripheral axons, several pivotal miRNAs exist, amenable to post-transcriptional regulation, thereby exerting a profound influence on the intricate process of axon regeneration [52]. The miRNA-9 is one of the most abundant miRNAs in the developing and adult brain [36]. In the early years, researchers focused on the role of miRNA-9 in tumors and cancers, and then gradually studied in neural development and regeneration. Previous studies have found that miRNA-9 affects the migration of Schwann cells by targeting Dcc [53]. It is also able to promote dendritic growth and synaptic transmission by down-regulating REST [36]. Nevertheless, these results were obtained in cell populations, and their effects at the single-cell level have yet to be comprehensively elucidated. In this study, we investigated the role of miRNA-9 in CNS axon regeneration by manipulating its expression at the single-cell level using the zebrafish Mauthner single-cell axon regeneration model. To the best of our knowledge, miRNA-9 can promote intracellular calcium activity in neurons by inhibiting the expression of its downstream target gene *her6*, which in turn promotes axonal regeneration.

As mentioned above, research on miRNA-9 has been going on for many years. Extensive research has brought to light the multifaceted role of miRNA-9, such as overexpression of miRNA-9 inhibits Schwann cell migration in cultured cells in vitro [53, 54], promotes neuronal differentiation in mice embryos and adult NPCs [14, 37, 55], has anti-apoptosis effects in rat ASCI (Acute Spinal Cord Injury) models [56], controls axon extension and development [16, 17, 36] and inhibits axon regeneration in peripheral sensory neurons [17]. In short, a growing consensus that miRNA-9 has a certain neuroprotective effect. Our results demonstrate that miRNA-9 promotes axon regeneration in the zebrafish CNS, which is in contrast to the results of peripheral axonal regeneration, and we have three points to address this discrepancy. First, these two results are in the CNS and the PNS, respectively. We all know that there are differences in the regeneration ability of axons themselves in these two systems. Previous studies have also found that miRNAs can play different or even opposite roles in different tissues by regulating different targets [9]. Even at different time points, miRNA-9 has different effects on the same cell—neural progenitor cells [14, 37, 57]. Second, the previous peripheral results were conducted on neurons cultured in vitro when the neurons were in the developmental stage, whereas our experiments were observed in vivo, when the neurons were in the regeneration stage. The differences between growth stages and in vivo and in vitro also mean that miRNA may play different functions. Third, through a large amount of miRNA high-throughput sequencing data, we can also see that miRNA-135a, which is consistent with the expression trend of miRNA-9 after injury, also shows a strong promoting effect in axon regeneration, which has certain reference significance for our study on the role of miRNA-9 in axon regeneration.

In this experiment, we used CRISPR-Cas9 to knock out miRNA-9 in zebrafish. We found that due to the short length of miRNA-9 (only 22 nt), it could only be performed on pri-miRNA-9 when designing the target. After designing the knockout targets, the construction of knockout lines was carried out. During the construction process, the 16 bp base sequence was deleted, but homozygote and knockout zebrafish could not be obtained. This implies that miRNA-9 not only plays a role in neural development, but also has an important function in zebrafish survival and development. The target of miRNA-9 deletion in previous studies was not successfully knocked out in this experiment due to the self-repair of the bases. Different knockout targets can lead to different degrees of gene deletion, which may have different effects on the viability of zebrafish larvae. Experiments conducted in miRNA-9^±^ zebrafish strain indicated heterozygous knockout that did not affect axonal regeneration and did not cause adverse effects on the development of axon and overall larvae growth (Fig. S2). Whereas the specific suppression of miRNA-9 expression within a single cell notably hindered axon regeneration in zebrafish M-cells (Fig. 1h, j). The discrepancy in results could be attributed to the differences in the level and specificity of miRNA-9 manipulation between the two experimental approaches. The heterozygote knockout perturbed the overall miRNA-9 expression, possibly exerting a mild or insufficient effect to substantially alter axon regeneration due to the presence of the normal allele and the dose effect did not reach the threshold. Conversely, the targeted suppression in a single cell could produce a more precise and substantial impact on axon regeneration owing to the localized and focused inhibition of miRNA-9 expression, thereby showcasing a clearer phenotype.

When it comes to the downstream of miRNA-9, most studies have focused on miRNA-9 regulating members of *her* family and affecting neurogenesis in the zebrafish brain, which is consistent with the results of miR-9 targeting of mice *HES1*. Most of them are proved to be downstream targets of miRNA-9 utilizing ISH and immunofluorescence, such as *her5*, *her6*, and *her9* [41, 44, 58]. Whereas, in this study, we used in vivo imaging with EGFP-sensor to provide compelling evidence that *her6* is a downstream target gene of miRNA-9. Due to limitations related to the availability of zebrafish antibodies, we regrettably could not assess alterations in Her6 at the protein level following the manipulation of miRNA-9 expression. However, based on the results of in vivo imaging and qPCR, we believe that such evidence is also convincing. As stated above, most of the studies on *her6* in zebrafish have focused on the role of brain neurogenesis, while *HES1* in mice has been studied in a wide range of neurogenesis, immunity, and tumors [59, 60], but not involve its function in axon regeneration. Surprisingly, this study stands as the inaugural account to establish a definitive connection between *her6* and axon regeneration, shedding light on the previously uncharted inhibitory role of *her6* in CNS axon regeneration. On the other hand, the zebrafish strain with *her6* knockout proved that there was no defect in body development and axon development, and the function of promoting regeneration after *her6* knockout was completed by affecting the endogenous factors of axons (Fig. 4).

In the initial stage of axonal injury, the influx of Ca^2+^ provides a damage signal, which has a key impact on whether axons can regenerate later [28, 61]. He et al. [3], when discussing the intrinsic factors affecting nerve regeneration, proposed that manipulating neuronal activity may be related to enhancing the intrinsic growth ability of neurons, and this hypothesis was also proved in the study of Chen et al. [21]. The gene *Cacna2d2*, encoding the Alpha2delta2 subunit of the voltage-gated calcium channel (VGCC), has also been found to act as a developmental switch to limit axonal growth and generation of dorsal root ganglion (DRG) neurons in adult mice [62]. All these are suggestive of the relationship between neuronal activity and axonal regeneration. With the development of optical imaging technology and genetically encoded calcium indicators (GECI), two-photon calcium imaging (2PCI) has gradually become one of the commonly used indicators of neuronal activity. It is based on the principle that when a neuron excites an action potential, intracellular calcium levels increase, which can be detected by fluorescent molecules bound to calcium [26, 27]. Mauthner cell network, as one of the earliest circuits in the zebrafish brain studied using calcium imaging [29, 30, 63], also provided an important basis for our current study. The NEMOf used in this experiment has the advantage of being more sensitive and faster than the previous GCaMP series of calcium indicators, and the amplitude of the calcium peak is further higher than that of the Gcamp series under the same conditions. In addition, there are five types of NEMO indicators. In terms of a comprehensive comparison of imaging speed and imaging effect, NEMOf, which has medium calcium peak amplitude and the most rapid calcium indication, was selected [31]. In this study, we not only used pharmacological approaches to modify neuronal activity in zebrafish, but also manipulated gene expression within M-cells to observe the changes in calcium levels. It is worth mentioning that in the *her6*-overexpressing zebrafish, the introduction of PTZ treatment resulted in a remarkable elevation of calcium levels when compared to the *her6*-overexpressing group. This effect was accompanied by a remarkable restoration of axonal regeneration ability (Fig. 6).

In conclusion, our study investigated the role of miRNA-9 in the CNS axon regeneration at the single-cell level and found that miRNA-9 promoted the upregulation of neuronal calcium level through inhibiting the expression of its downstream target gene *her6*, thereby promoting axonal regeneration. Our study revealed a novel role of the post-transcriptional regulator miRNA-9 in the CNS that has not been reported and it is also expected that miRNA-9 can become a new target for the treatment of spinal cord injury.

### Supplementary Information

Below is the link to the electronic supplementary material.

Supplementary file1 (DOCX 42054 KB)

## Data Availability

All data generated or analyzed during this study are included in this published article and its supplementary information files.

## References

[1] TNSS Center (2016). Spinal cord injury (sci) 2016 facts and figures at a glance. J Spinal Cord Med.

[2] Hu X, Xu W, Ren Y, Wang Z, He X, Huang R, Ma B, Zhao J, Zhu R, Cheng L (2023). Spinal cord injury: molecular mechanisms and therapeutic interventions. Signal Transduction Targeted Therapy.

[3] He Z, Jin Y (2016). Intrinsic control of axon regeneration. Neuron.

[4] David S, Aguayo AJ (1981). Axonal elongation into peripheral nervous system “bridges” after central nervous system injury in adult rats. Science.

[5] Ewan EE, Avraham O, Carlin D, Gonçalves TM, Zhao G, Cavalli V (2021). Ascending dorsal column sensory neurons respond to spinal cord injury and downregulate genes related to lipid metabolism. Sci Rep-Uk.

[6] Hilton BJ, Husch A, Schaffran B, Lin T-C, Burnside ER, Dupraz S, Schelski M, Kim J, Müller JA, Schoch S, Imig C, Brose N, Bradke F (2022). An active vesicle priming machinery suppresses axon regeneration upon adult cns injury. Neuron.

[7] Wang X, Zhou T, Maynard GD, Terse PS, Cafferty WB, Kocsis JD, Strittmatter SM (2020). Nogo receptor decoy promotes recovery and corticospinal growth in non-human primate spinal cord injury. Brain.

[8] Li P, Teng Z-Q, Liu C-M (2016). Extrinsic and intrinsic regulation of axon regeneration by micrornas after spinal cord injury. Neural Plast.

[9] Borger A, Stadlmayr S, Haertinger M, Semmler L, Supper P, Millesi F, Radtke C (2022). How mirnas regulate Schwann cells during peripheral nerve regeneration—a systemic review. Int J Mol Sci.

[10] Chen L, Chuang M, Koorman T, Boxem M, Jin Y, Chisholm AD (2015). Axon injury triggers efa-6 mediated destabilization of axonal microtubules via tacc and doublecortin like kinase. Elife.

[11] Hellal F, Hurtado A, Ruschel J, Flynn KC, Laskowski CJ, Umlauf M, Kapitein LC, Strikis D, Lemmon V, Bixby J, Hoogenraad CC, Bradke F (2011). Microtubule stabilization reduces scarring and causes axon regeneration after spinal cord injury. Science.

[12] Friedman RC, Farh KK-H, Burge CB, Bartel DP (2009). Most mammalian mrnas are conserved targets of micrornas. Genome Res.

[13] Fiore R, Siegel G, Schratt G (2008). Microrna function in neuronal development, plasticity and disease. Biochimica et Biophysica Acta (BBA) Gene Regulatory Mechanisms.

[14] Yuva-Aydemir Y, Simkin A, Gascon E, Gao F-B (2014). Microrna-9 functional evolution of a conserved small regulatory rna. RNA Biol.

[15] Gong C-X, Yu B, Zhou S, Wang Y, Ding G, Ding F, Gu X (2011). Profile of micrornas following rat sciatic nerve injury by deep sequencing: Implication for mechanisms of nerve regeneration. PLoS ONE.

[16] Dajas-Bailador F, Bonev B, Garcez P, Stanley P, Guillemot F, Papalopulu N (2012). Microrna-9 regulates axon extension and branching by targeting map1b in mouse cortical neurons. Nat Neurosci.

[17] Jiang J, Hu Y, Zhang B, Shi Y, Zhang J, Wu X, Yao P (2017). Microrna-9 regulates mammalian axon regeneration in peripheral nerve injury. Mol Pain.

[18] Wang N, Yang Y, Pang M, Du C, Chen Y, Li S, Tian Z, Feng F, Wang Y, Chen Z, Liu B, Rong L (2020). Microrna-135a-5p promotes the functional recovery of spinal cord injury by targeting sp1 and rock. Mol Therapy Nucleic Acids.

[19] Becker T, Becker CG (2014). Axonal regeneration in zebrafish. Curr Opin Neurobiol.

[20] Hu B-B, Chen M, Huang R-C, Huang Y-B, Xu Y, Yin W, Li L, Hu B (2018). In vivo imaging of mauthner axon regeneration, remyelination and synapses re-establishment after laser axotomy in zebrafish larvae. Exp Neurol.

[21] Chen M, Huang R-C, Yang L-Q, Ren D-L, Hu B (2019). In vivo imaging of evoked calcium responses indicates the intrinsic axonal regenerative capacity of zebrafish. FASEB J.

[22] Huang R, Chen M, Yang L, Wagle M, Guo S, Hu B (2017). Microrna-133b negatively regulates zebrafish single mauthner-cell axon regeneration through targeting tppp3 in vivo. Front Mol Neurosci.

[23] Xu Y, Chen M, Hu B, Huang R, Hu B (2017). In vivo imaging of mitochondrial transport in single-axon regeneration of zebrafish Mauthner cells. Front Cell Neurosci.

[24] Yang L-Q, Chen M, Ren D-L, Hu B (2020). Dual oxidase mutant retards mauthner-cell axon regeneration at an early stage via modulating mitochondrial dynamics in zebrafish. Neurosci Bull.

[25] Zhang Y, Rózsa M, Liang Y, Bushey D, Wei Z, Zheng J, Reep D, Broussard GJ, Tsang A, Tsegaye G, Narayan S, Obara CJ, Lim J-X, Patel R, Zhang R, Ahrens MB, Turner GC, Wang SSH, Korff WL, Schreiter ER, Svoboda K, Hasseman JP, Kolb I, Looger LL (2023). Fast and sensitive gcamp calcium indicators for imaging neural populations. Nature.

[26] Grienberger C, Giovannucci A, Zeiger W, Portera-Cailliau C (2022). Two-photon calcium imaging of neuronal activity. Nat Rev Methods Primers.

[27] Tort-Colet N, Resta F, Montagni E, Pavone F, Allegra Mascaro AL, Destexhe A (2023). Assessing brain state and anesthesia level with two-photon calcium signals. Sci Rep-Uk.

[28] Huang C-X, Zhao Y, Mao J, Wang Z, Xu L, Cheng J, Guan NN, Song J (2021). An injury-induced serotonergic neuron subpopulation contributes to axon regrowth and function restoration after spinal cord injury in zebrafish. Nat Commun.

[29] Kettunen P (2020). Calcium imaging in the zebrafish. Adv Exp Med Biol.

[30] Alix MB, Lacoste DS, Robson DN, Haesemeyer M, Portugues R, Li JM, Randlett O, Wee CL, Engert F, Schier AF (2015). A convergent and essential interneuron pathway for mauthner-cell-mediated escapes. Curr Biol.

[31] Li J, Shang Z, Chen J-H, Gu W, Yao L, Yang X, Sun X, Wang L, Wang T, Liu S, Li J, Hou T, Xing D, Gill DL, Li J, Wang S-Q, Hou L, Zhou Y, Tang A-H, Zhang X, Wang Y (2023). Engineering of nemo as calcium indicators with large dynamics and high sensitivity. Nat Methods.

[32] Dong Z, Peng J, Guo S (2013). Stable gene silencing in zebrafish with spatiotemporally targetable rna interference. Genetics.

[33] De Rienzo G, Gutzman JH, Sive H (2012). Efficient shrna-mediated inhibition of gene expression in zebrafish. Zebrafish.

[34] Rongchen Huang YX, Chen M, Yang L, Wang X, Yueru Shen YH, Hu B (2022) Visualizing the intracellular trafficking in zebrafish mauthner cells. Methods Mol Biol 10.1007/978-1-0716-1990-2_18 35412286

[35] Sagasti A, O’Brien GS, Rieger S, Martin SM, Cavanaugh AM, Portera-Cailliau C (2009) Two-photon axotomy and time-lapse confocal imaging in live zebrafish embryos. J Vis Exp 10.3791/1129 PMC2726581 19229185

[36] Giusti SA, Vogl AM, Brockmann MM, Vercelli CA, Rein ML, Trümbach D, Wurst W, Cazalla D, Stein V, Deussing JM, Refojo D (2014). Microrna-9 controls dendritic development by targeting rest. Elife.

[37] Coolen M, Katz S, Bally-Cuif L (2013). Mir-9: a versatile regulator of neurogenesis. Front Cell Neurosci.

[38] ErnoWienholds WP, Kloosterman EM, Alvarez-Saavedra E, Berezikov E, Ewart de Bruijn H, Horvitz R, Kauppinen S, Plasterk RHA (2005). Microrna expression in zebrafishembryonic development. Science.

[39] Alkan AH, Akgul B (2022). Endogenous mirna sponges. Methods Mol Biol.

[40] Cohen SM (2009). Use of microrna sponges to explore tissue-specific microrna functions in vivo. Nat Methods.

[41] Scholpp S, Delogu A, Gilthorpe J, Peukert D, Schindler S, Lumsden A (2009). Her6 regulates the neurogenetic gradient and neuronal identity in the thalamus. Proc Natl Acad Sci.

[42] Soto X, Biga V, Kursawe J, Lea R, Doostdar P, Thomas R, Papalopulu N (2020). Dynamic properties of noise and her6 levels are optimized by mir-9, allowing the decoding of the her6 oscillator. The EMBO J.

[43] Giraldez AJ, Cinalli RM, Glasner ME, Enright AJ, Thomson JM, Baskerville S, Hammond SM, Bartel DP, Schier AF (2005). Micrornas regulate brain morphogenesis in zebrafish. Science.

[44] Leucht C, Stigloher C, Wizenmann A, Klafke R, Folchert A, Bally-Cuif L (2008). Microrna-9 directs late organizer activity of the midbrain-hindbrain boundary. Nat Neurosci.

[45] Morgan JI, Curran T (1988). Calcium as a modulator of the immediate-early gene cascade in neurons. Cell Calcium.

[46] Cho SJ, Park E, Telliyan T, Baker A, Reid AY (2020). Zebrafish model of posttraumatic epilepsy. Epilepsia.

[47] Berdyyeva TK, Frady EP, Nassi JJ, Aluisio L, Cherkas Y, Otte S, Wyatt RM, Dugovic C, Ghosh KK, Schnitzer MJ, Lovenberg T, Bonaventure P (2016). Direct imaging of hippocampal epileptiform calcium motifs following kainic acid administration in freely behaving mice. Front Neurosci.

[48] Zwaka H, McGinnis OJ, Pflitsch P, Prabha S, Mansinghka V, Engert F, Bolton AD (2022). Visual object detection biases escape trajectories following acoustic startle in larval zebrafish. Curr Biol.

[49] Xu L, Guan NN, Huang C-X, Hua Y, Song J (2021). A neuronal circuit that generates the temporal motor sequence for the defensive response in zebrafish larvae. Curr Biol.

[50] Lasseigne AM, Echeverry FA, Ijaz S, Michel JC, Martin EA, Marsh AJ, Trujillo E, Marsden KC, Pereda AE, Miller AC (2021). Electrical synaptic transmission requires a postsynaptic scaffolding protein. Elife.

[51] Hecker A, Schulze W, Oster J, Richter DO, Schuster S (2020). Removing a single neuron in a vertebrate brain forever abolishes an essential behavior. Proc Natl Acad Sci.

[52] Wu D, Raafat A, Pak E, Clemens S, Murashov AK (2012). Dicer-microrna pathway is critical for peripheral nerve regeneration and functional recovery in vivo and regenerative axonogenesis in vitro. Exp Neurol.

[53] Wang X, Chen Q, Yi S, Liu Q, Zhang R, Wang P, Qian T, Li S (2019). The micrornas let-7 and mir-9 down-regulate the axon-guidance genes ntn1 and dcc during peripheral nerve regeneration. J Biol Chem.

[54] Zhou S, Gao R, Hu W, Qian T, Wang N, Ding G, Ding F, Yu B, Gu X (2014). Mir-9 inhibits Schwann cell migration by targeting cthrc1 following sciatic nerve injury. J Cell Sci.

[55] Radhakrishnan B, Anand AAP (2016). Role of mirna-9 in brain development. J Exp Neurosci.

[56] Wu J, Li H, He J, Tian X, Luo S, Li J, Li W, Zhong J, Zhang H, Huang Z, Sun X, Jiang T (2021). Downregulation of microrna-9-5p promotes synaptic remodeling in the chronic phase after traumatic brain injury. Cell Death Dis.

[57] Bonev B, Pisco A, Papalopulu N (2011). Microrna-9 reveals regional diversity of neural progenitors along the anterior-posterior axis. Dev Cell.

[58] Dirian L (2014) Embryonic origin of adult neural stem cells in the zebrafish pallium. Journal

[59] Dhanesh SB, Subashini C, James J (2016). Hes1: the maestro in neurogenesis. Cell Mol Life Sci.

[60] Rani A, Greenlaw R, Smith RA, Galustian C (2016). Hes1 in immunity and cancer. Cytokine Growth Factor Rev.

[61] Sun F, He Z (2010). Neuronal intrinsic barriers for axon regeneration in the adult cns. Curr Opin Neurobiol.

[62] Tedeschi A, Dupraz S, Laskowski CJ, Xue J, Ulas T, Beyer M, Schultze JL, Bradke F (2016). The calcium channel subunit alpha2delta2 suppresses axon regeneration in the adult cns. Neuron.

[63] Fetcho JR, O’Malley DM (1995). Visualization of active neural circuitry in the spinal cord of intact zebrafish. J Neurophysiol.

