# Supplementary material for: MicroRNA-9 promotes axon regeneration of mauthner-cell in zebrafish via her6/ calcium activity pathway
Source: Cell Mol Life Sci. 2024 Feb 27;81(1):104. doi: 10.1007/s00018-024-05117-2 (PMC10899279; doi:10.1007/s00018-024-05117-2)
Supplement: Supplementary file 1 — Supplementary file1 (DOCX 42054 KB) [file 18_2024_5117_MOESM1_ESM.docx]

**MicroRNA-9 Promotes Axon Regeneration of Mauthner-Cell in Zebrafish via *her6*/ Calcium Activity Pathway**

Yueru Shen^1^, Xinghan Chen^1^, Song Zheng^1^, Huaitong Yao^1^, Along Han^1^, Yawen Zhang^1^, Yuan Cai^3^, Bing Hu^1,2*^

^1^Hefei National Research Center for Physical Sciences at the Microscale, University of Science and Technology of China, Hefei 230026, China

^2^Center for Advanced Interdisciplinary Science and Biomedicine of IHM, Division of Life Sciences and Medicine, University of Science and Technology of China, Hefei 230026, China,

^3^First Affiliated Hospital of USTC, Division of Life Sciences and Medicine, University of Science and Technology of China, Hefei 230026, China

*Correspondence: [bhu@ustc.edu.cn](mailto:bhu@ustc.edu.cn)

ORCID: 0000-0001-7670-6243 (Yueru Shen), 0000-0001-8627-5272 (Bing Hu)


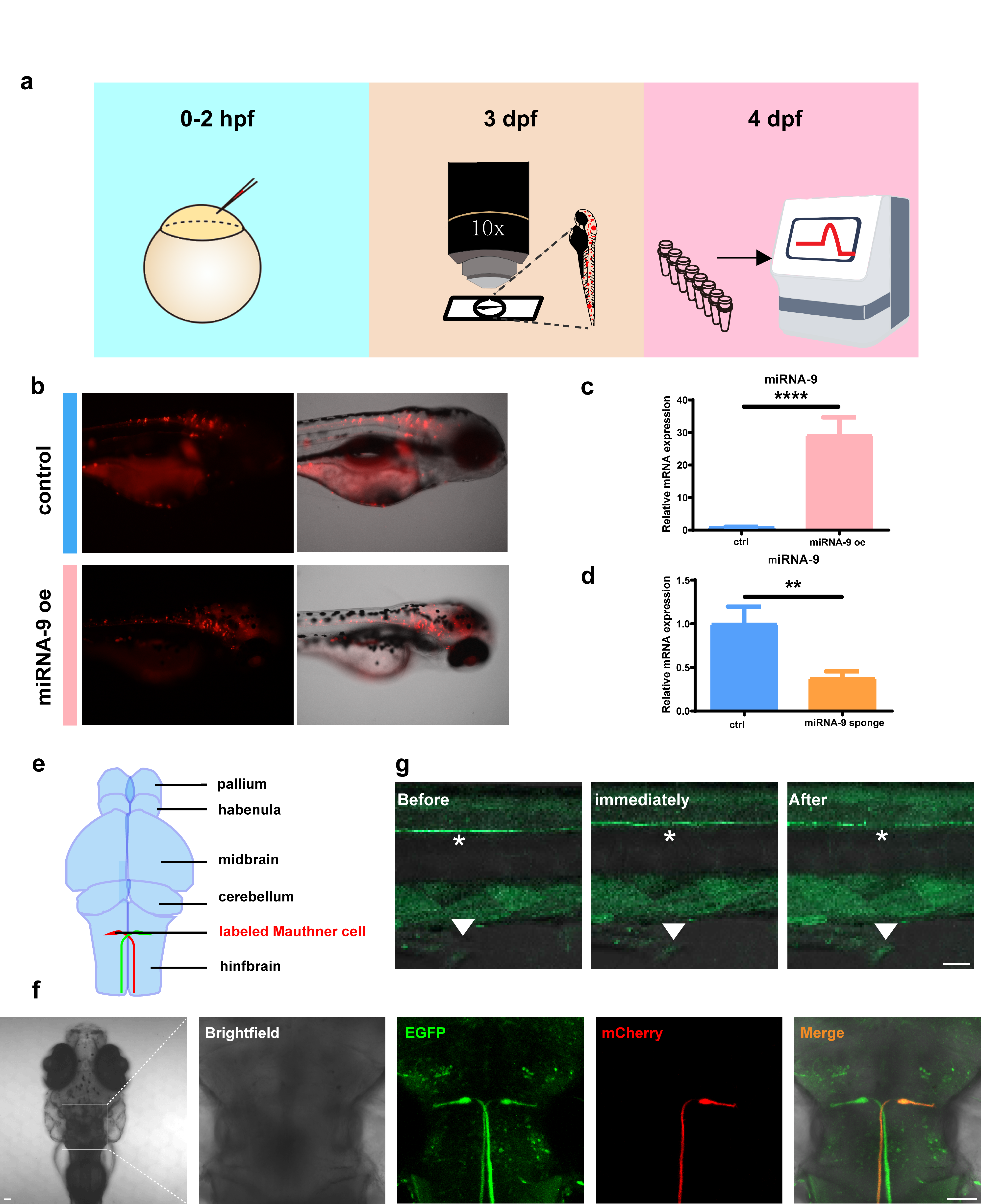


**Fig. S1** Validation of the effectiveness of the overexpression plasmid using microinjection and electroporation. (a) Timeline of time points of microinjection, screening and Quantitative RT-PCR. (b) Representative images of the central nervous system with red fluorescence expression. The Left is the image under the mCherry fluorescence, and the right is the merge of the brightfield and mCherry. (c) Quantitative RT-PCR analysis exhibited the plasmid of miRNA 9 oe increased the expression level of miRNA 9 in 4 dpf zebrafish (control: 1.0 ± 0.0417; miRNA 9 oe: 29.17 ± 5.51, n = 3). *p* < 0.0001, assessed by t-test. (d) Quantitative RT-PCR analysis exhibited a decrease in miRNA 9 expression in 4 dpf zebrafish larvae by the vector-based miRNA 9 sponge *in vivo*. *p* = 0.0098. Assessed by unpaired t-test. (e) Schematic of M-cell soma electroporation. (f) Confocal imaging of zebrafish larvae 24 h after electroporation (far left) and magnified images of the brain in zebrafish larvae, denoting the position of M-cell soma under different fluorescence in the white box. mCherry represents the axons that were labeled by mCherry. Scale bar, 50 μm. (g) Representative images of the M-cell axon before and after ablations by a two-photon laser. Asterisk, injury site; arrowhead, cloacal pore; scale bar, 50 μm.


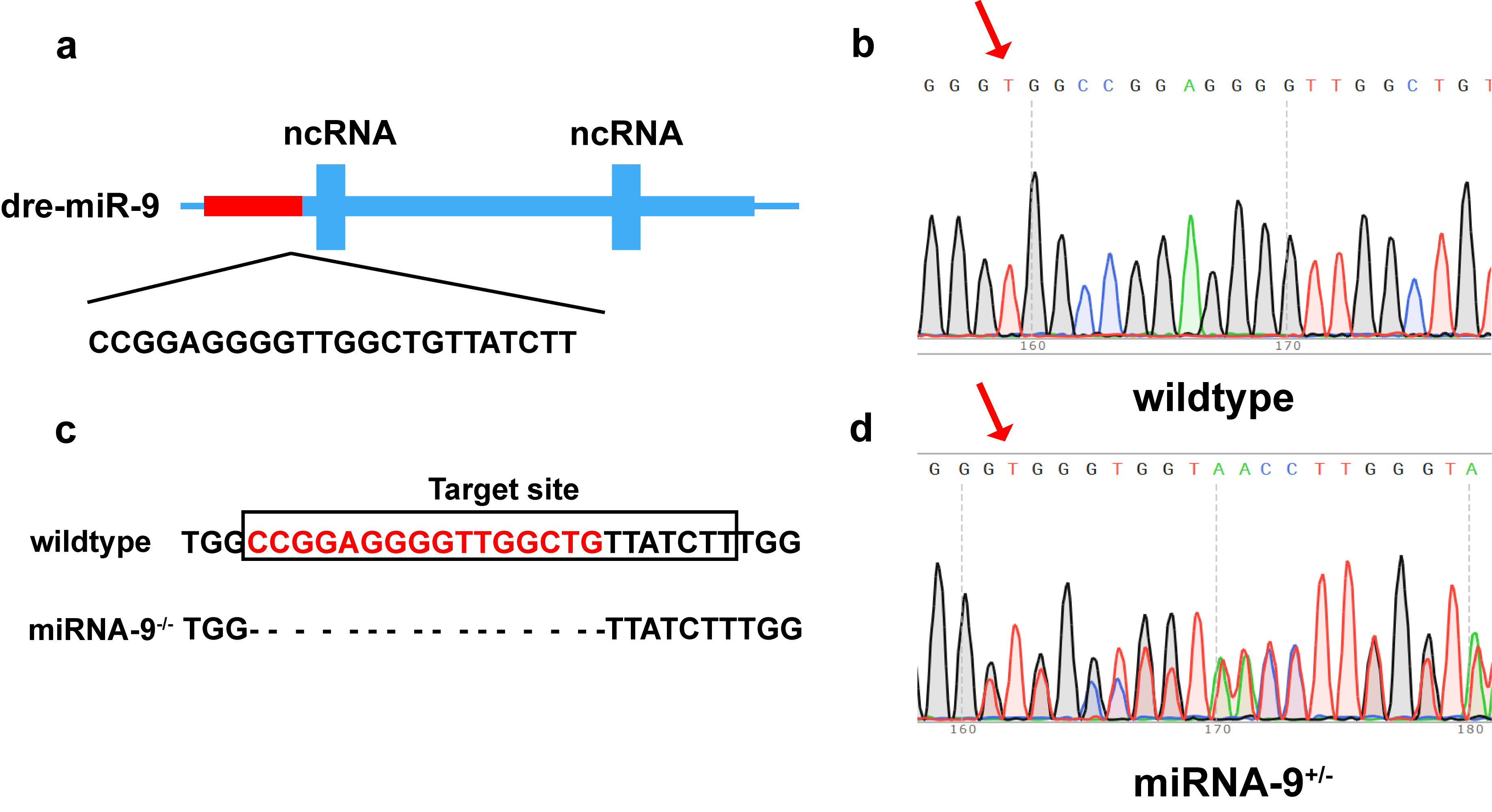


**Fig. S2** Generation of miRNA 9 knockout zebrafish using CRISPR/Cas9. (a) Schematic showing the Cas9-sgRNA-targeted site of the dre-miR-9. (b-d) Representative sequencing results of wide-type and mutated zebrafish lines. The sequence marked in red in (c) is a 16 bp deletion in the mutant zebrafish. The mutation start site is indicated by the red arrow.

.


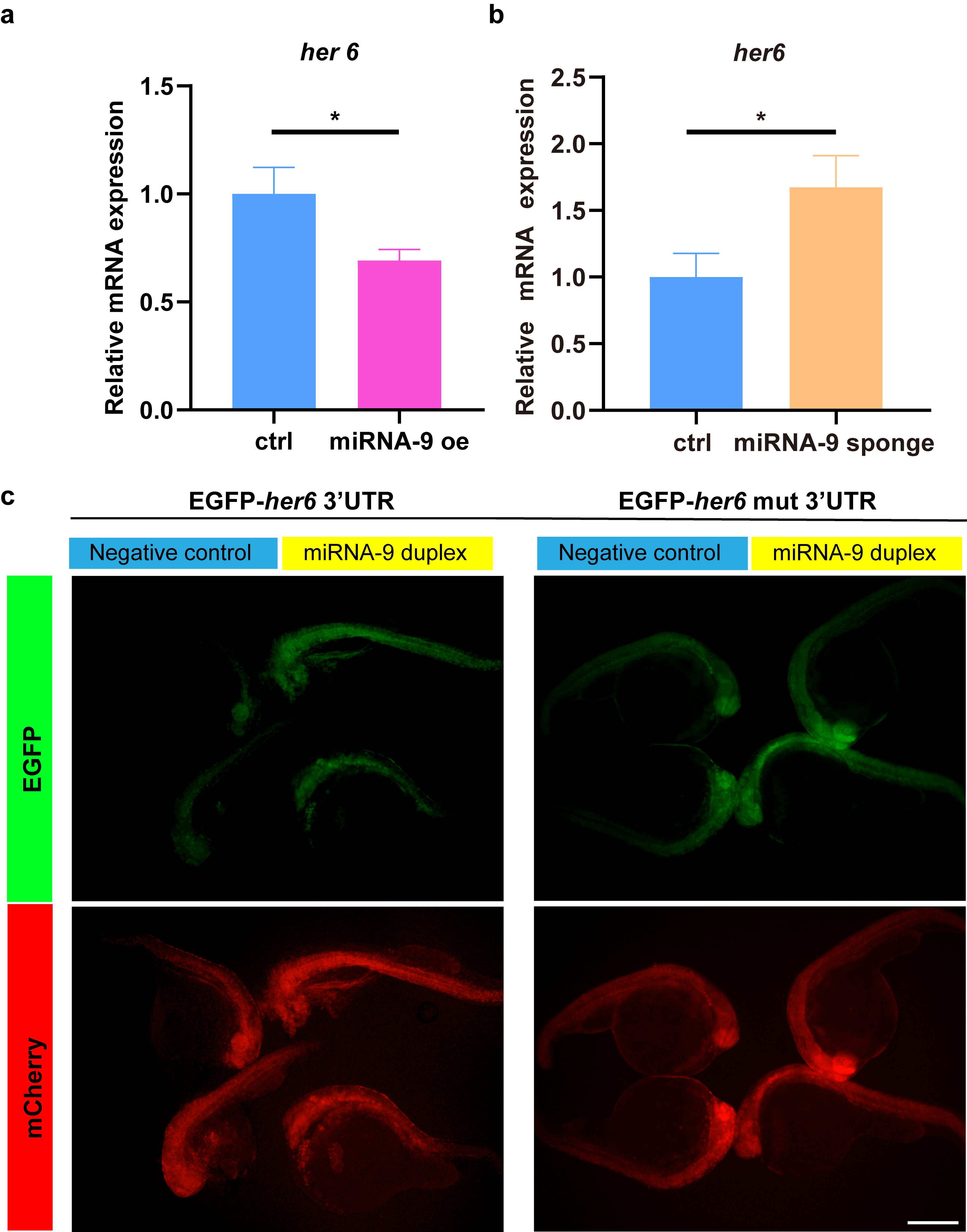


**Fig. S3** qPCR and the EGFP sensor assay show that *her6* is the downstream target of miRNA-9. (a) Quantitative RT-PCR analysis exhibited the plasmid of miRNA 9 oe inhibited the expression level of *her6* in 4dpf zebrafish (control: 1.000 ± 0.1226; miRNA 9 oe: 0.6914 ± 0.0522, n = 3). *p* = 0.0303, assessed by t-test. (b) Quantitative RT-PCR analysis exhibited the plasmid of miRNA 9 sponge increased the expression level of *her6* in 4dpf zebrafish (control: 1.000 ± 0.1773; miRNA 9 sponge: 1.674 ± 0.2381, n = 3). *p* = 0.0374, assessed by t-test. (c) EGFP sensor showed that *her6* is the downstream target of miRNA-9. EGFP-*her6* 3′UTR showed strong fluorescent signals when co-injected with non-sense duplex (as negative control), but failed to give fluorescent signals when co-injected with miRNA-9 duplex, and mCherry mRNA was injected as a control (left). EGFP-*her6* 3′UTR mut showed strong fluorescent signals both in non-sense duplex and miRNA-9 duplex (right).


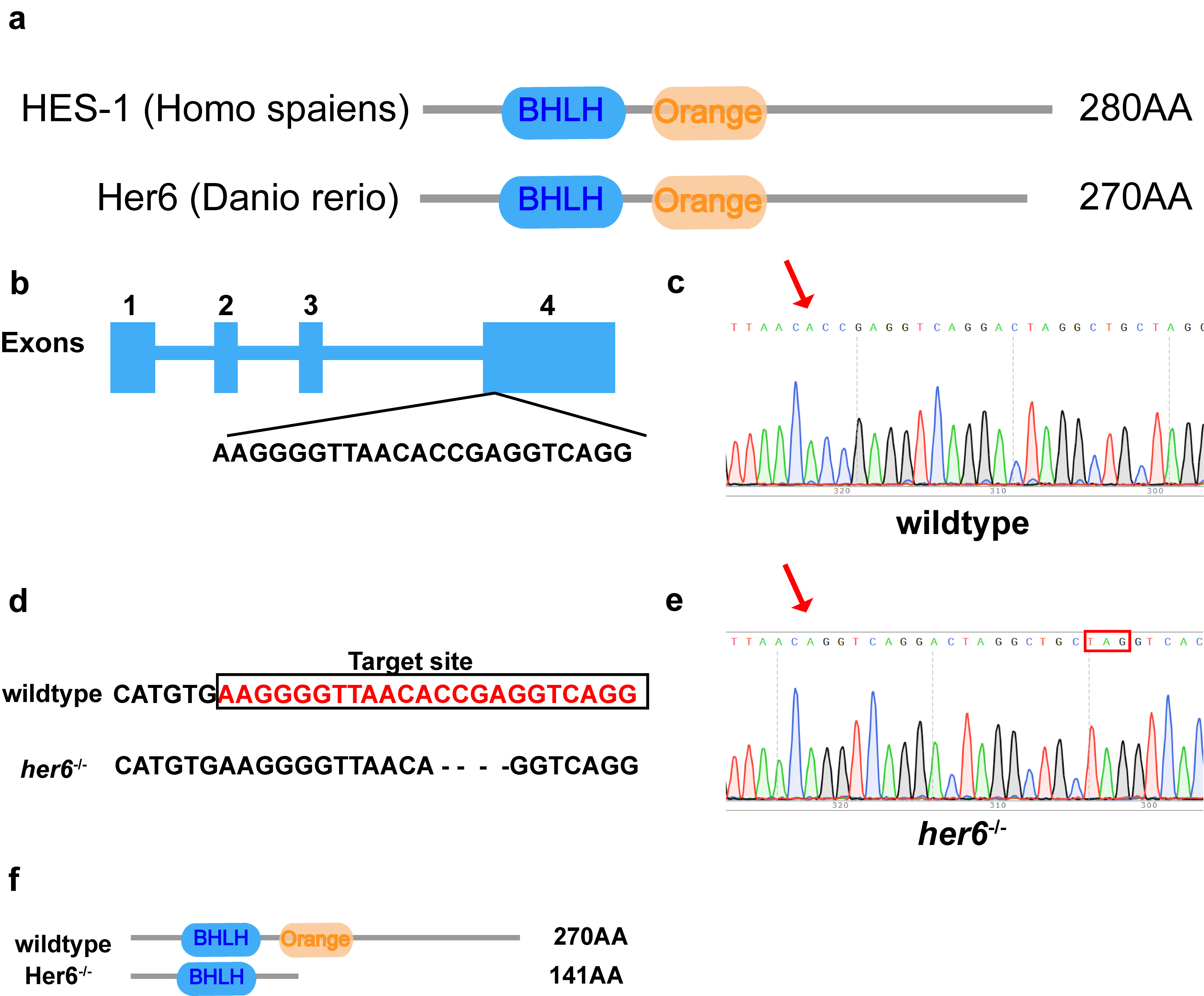


**Fig. S4** Generation of *her6* knockout zebrafish using CRISPR/Cas9. (a) The predicted protein structure of HES-1 in humans and Her6 in zebrafish. (b) Schematic showing the Cas9-sgRNA-targeted site located in the fourth exon of the *her6* gene. (c-e) Representative sequencing results of wide-type and mutated zebrafish lines. The sequence marked in red in (d) is a 4 bp deletion in the mutant zebrafish. The mutation start site (c, e) is indicated by the red arrow. The termination codons (e) are boxed in red. (f) Bioinformatics analysis indicated that the mutated region is locate in the orange domain of Her6. The wildtype translated to 270 amino acids, whereas the mutant translated to 141 normal amino acids.


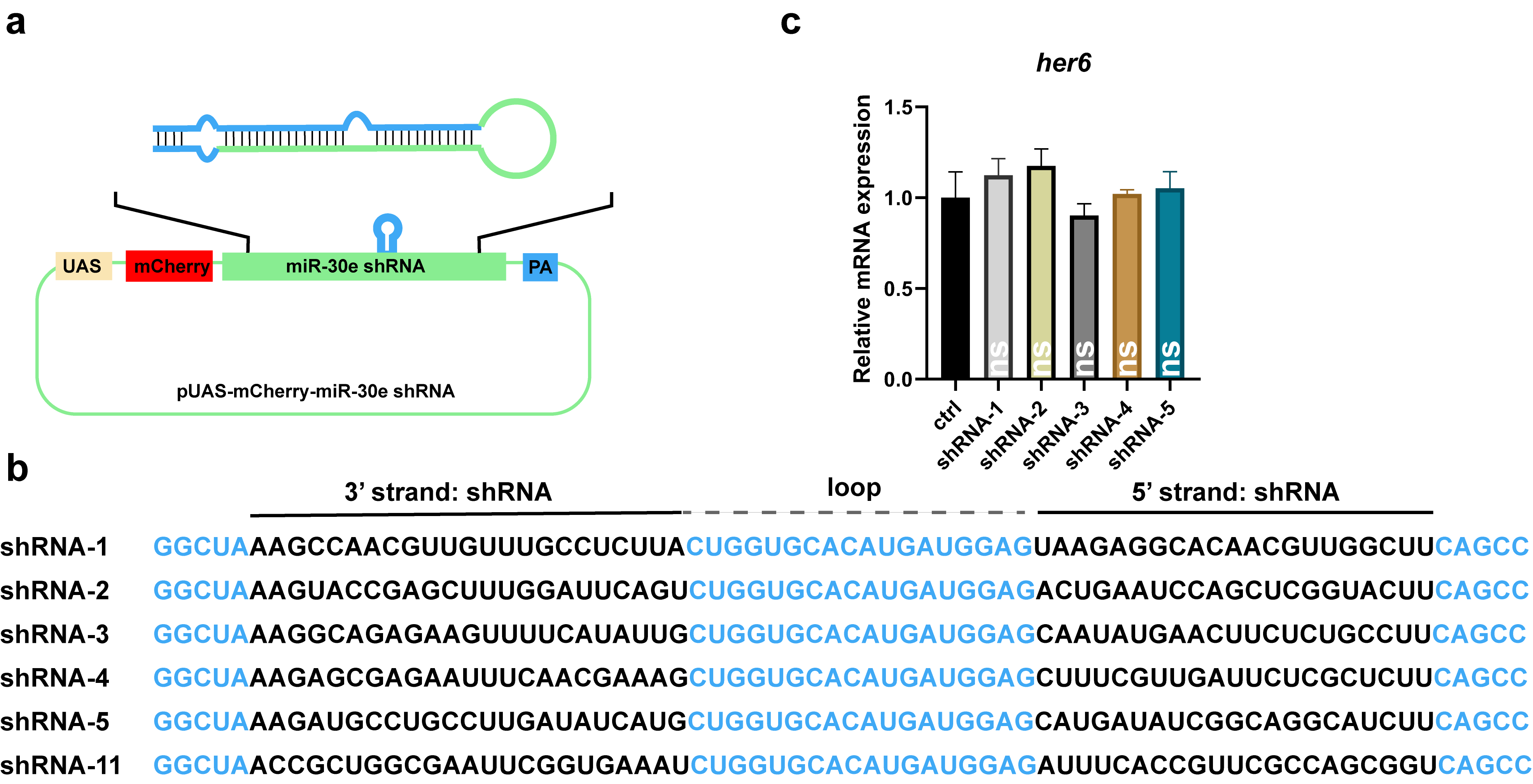


**Fig. S5** Design and screening of *her6* shRNA. (a) Design of the *her6* shRNA expression system based on the miR-30e backbone. (b) Sequence comparison of different shRNAs. (c) Quantitative statistical diagram of knockdown efficiency of target genes *her6* by shRNA. ctrl vs shRNA-1: *p* = 0.4762; ctrl vs shRNA-2: *p* = 0.3206; ctrl vs shRNA-3: *p* = 0.5386; ctrl vs shRNA-4: *p* = 0.9367; ctrl vs shRNA-5: *p* = 0.7627. Assessed by student’s two-tailed t-test. ns, not significant.


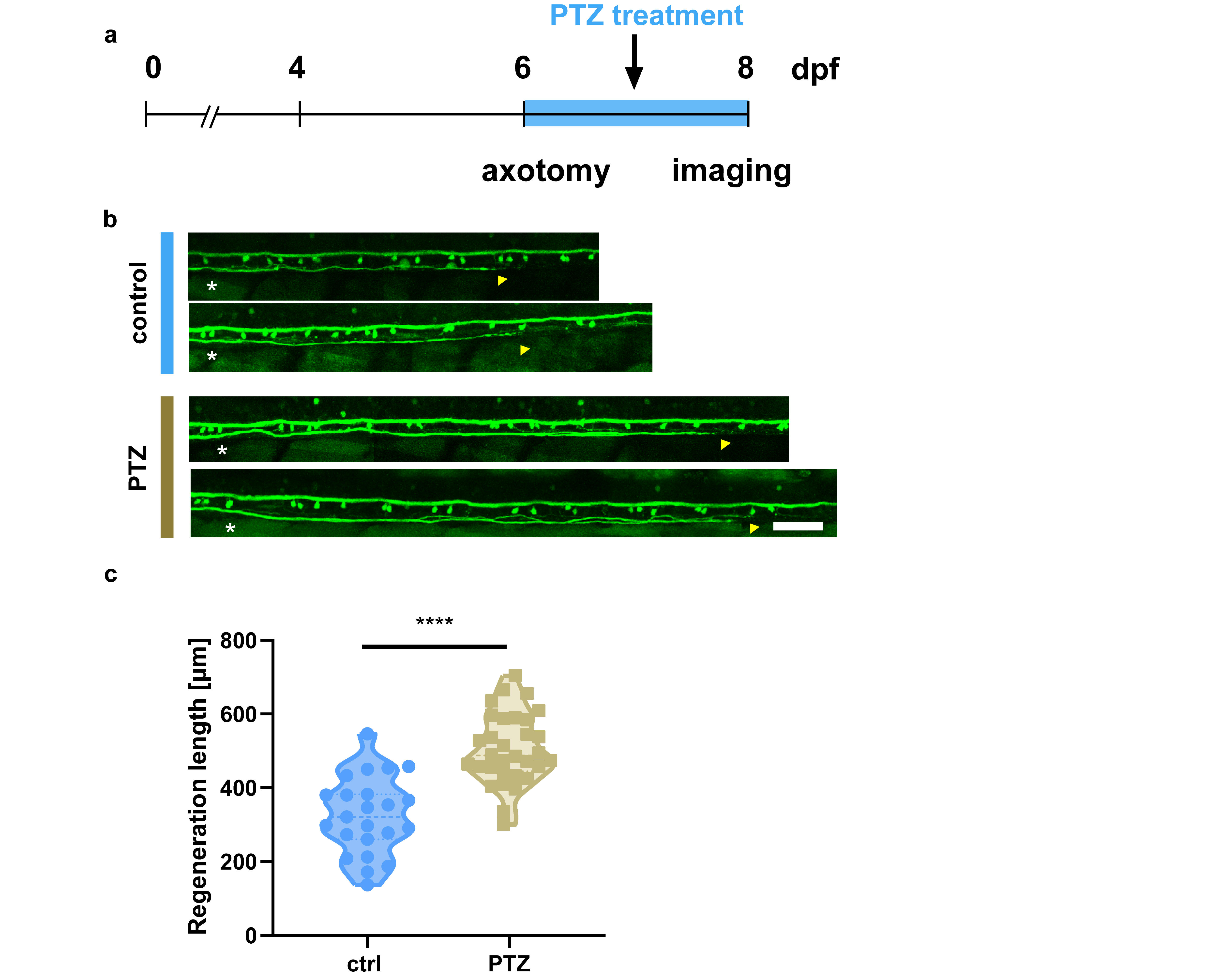


**Fig. S6** PTZ treatment increased the axonal regeneration ability. (a) The time axis showed the time-points of axotomy, PTZ treatment, and regeneration imaging. (b, c) PTZ treatment promotes M-cells axon regeneration (control: 325.5 ± 21.72 μm, n = 23 fish; PTZ: 505.0 ± 16.76 μm, n = 32 fish). White asterisk: ablation point. scale bar, 50 μm. *p* < 0.0001. Assessed by student’s two-tailed t-test.


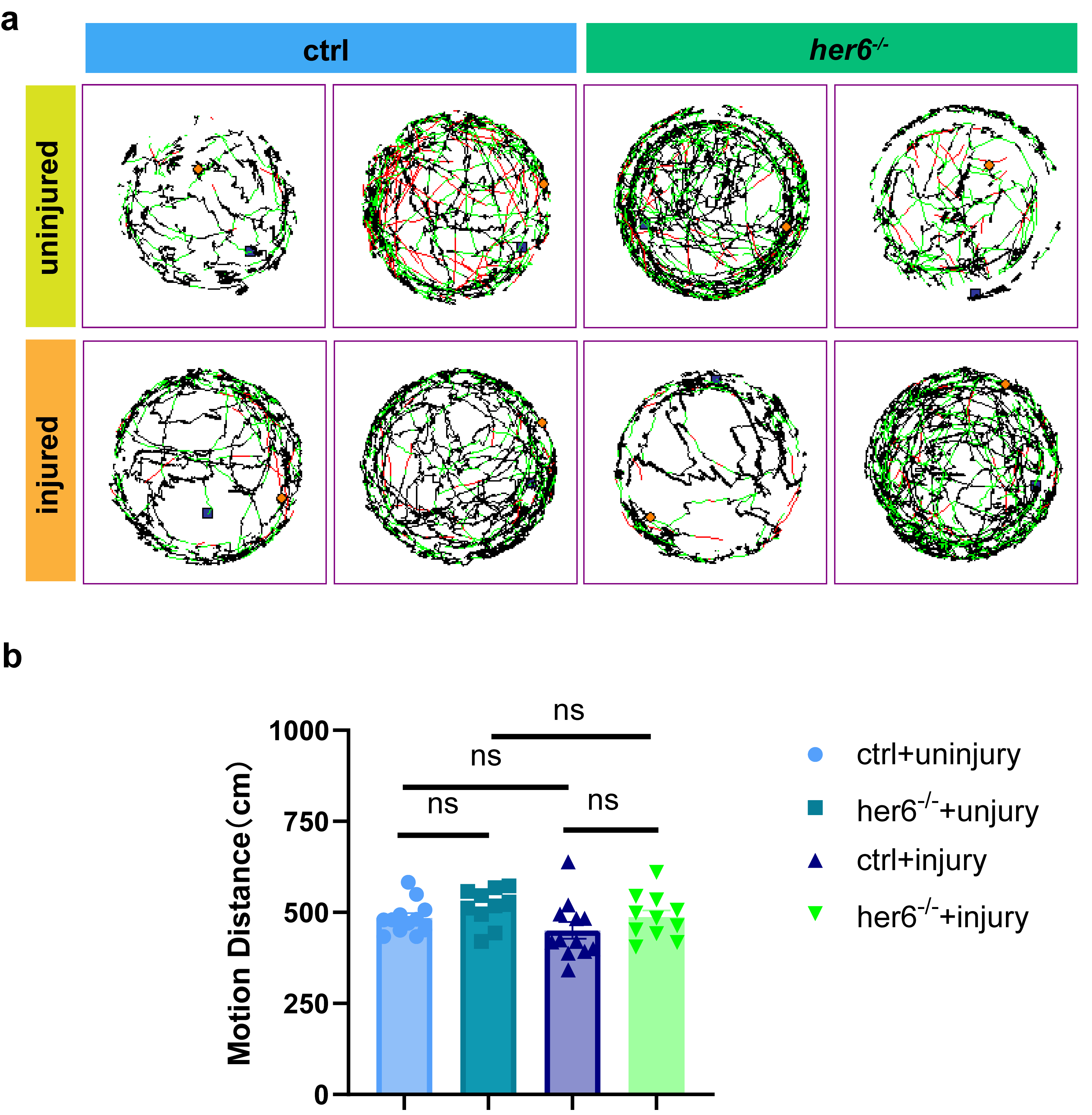


**Fig. S7** *her6^-/-^* zebrafish had no notably difference in free-swimming behavior after axotomy at 8 dpf. (a-b) Trajectory diagrams and motion distance statistics of free swimming within 1 hour of *her6*^-/-^ after axotomy (control + uninjured: 484.6 ± 12.96 cm, n = 12 fish; *her6*^-/-^+ uninjured: 516.3 ± 16.20 cm, n = 10 fish; control + injured: 450.7 ± 22.78 cm, n = 12 fish; *her6*^-/-^+ injured: 487.4 ± 18.19 cm, n = 11 fish). Assessed by one-way ANOVA. ns, not significant.

**Supplementary Table 1 Primers sequences for this study**

| Name | Primer sequences (5’->3’) | Usage |
| --- | --- | --- |
| miR-9 oe-F | AAATAAATCCCAGCCTTTGCGG | amplification |
| miR-9 oe-R | GTGGGGTGATGCCTTTCAGG | amplification |
| *her6* oe-F | CTGTACAAGTAGGCGGCCGCCCCAGAGAAGATGCCTGCCG | amplification |
| *her6* oe-R | TGATCTAGAGTCGCGGCCGCCCAAGGCCGCCAAACGGAGT | amplification |
| shRNA-30e-F | GAGCTCCTCCACACGAATTCACAGCCATGCCATAGTTTTAGG | amplification |
| shRNA-30e-R | TCCTCGCCCTTGCTCACCATATGAGTTCATCATATGACCAGTGAC | amplification |
| shRNA-30e-linker-F | GGCAGTACGGGCTACCCGGGAGCCAACTGCTGTTACTCTC | amplification |
| shRNA-30e-linker-R | AACAGCAGTTGGCTCCCGGGTAGCCCGTACTGCCAGCTG | amplification |
| *her6*-shRNA-11-F | CAGTACGGGCTACCCGGCTAACCGCTGGCGAATTCGGTGAAATCTGGTGCACATGATGG | amplification |
| *her6*-shRNA-11-R | CAGCAGTTGGCTCCCGGCTGACCGCTGGCGAACGGTGAAATCTCCATCATGTGCACCAG | amplification |
| miR-9 sg-F | GGACGGGTGGCCGGAGGGGT | sgRNA template |
| miR-9 sg-R | AGCACCGACTCGGTGCCACT | sgRNA template |
| Her6 sg-F | AAGGGGTTAACACCGAGGTC | sgRNA template |
| Her6 sg-R | AGCACCGACTCGGTGCCACT | sgRNA template |
| miR-9 KO-F | TGGATGGAAATGGACGGGTG | KO test |
| miR-9 KO-R | GGAGAAGAGGAGAGGGGAGG | KO test |
| Her6 KO-F | GTAAGTACCGAAAGTCCGACTGA | KO test |
| Her6 KO-R | GGAACCGGTGTGTTGGAATTG | KO test |
| *β-actin*-F | CCCTGTTCCAGCCATCCTT | qPCR |
| *β-actin*-R | TTGAAAGTGGTCTCGTGGATACC | qPCR |
| miR-9-F | GGGTTGGCTGTTATCTTTGGT | qPCR |
| miR-9-R | GGGATTCTTGTTACTTTCGGTTATC | qPCR |
| *her6*-F | GGCTTCGGAACACAGAAAGT | qPCR |
| *her6*-R | TGACCCAAGCTTTCGTTGA | qPCR |
| mCherry-F | TAATACGACTCACTATAGGGAGAGCCACCATGGTGAGCAAGGGCGAGGA | EGFP-sensor |
| mCherry-F | CTACTTGTACAGCTTGTCCAT | EGFP-sensor |
| EGFP-F | TAATACGACTCACTATAGGGAGAGCCACCATGGTGAGCAAGGGCGAG | EGFP-sensor |
| EGFP-R | CGCGGATCCTTACTTGTACAGCTCGTCCATGCC | EGFP-sensor |
| EFGP-*her6*-3’UTR-F | TCACGTCAGACTCCGTTTGG | EGFP-sensor |
| EFGP-*her6*-3’UTR-R | AAGAAGAAACTTGAAAGCGCATT | EGFP-sensor |
| EFGP-*her6*-3’UTR-mut-F | TCACGTCAGACTCCGTTTGG | EGFP-sensor |
| EFGP-*her6*-3’UTR-mut-R | AAGAAGAAACTTGAAAGCGCATTCAACATATGAAACCC | EGFP-sensor |
